# Midgut-derived neuropeptide F controls germline stem cell proliferation in a mating-dependent manner

**DOI:** 10.1371/journal.pbio.2005004

**Published:** 2018-09-24

**Authors:** Tomotsune Ameku, Yuto Yoshinari, Michael J. Texada, Shu Kondo, Kotaro Amezawa, Goro Yoshizaki, Yuko Shimada-Niwa, Ryusuke Niwa

**Affiliations:** 1 Graduate School of Life and Environmental Sciences, University of Tsukuba, Tsukuba, Japan; 2 Janelia Research Campus, Howard Hughes Medical Institute, Ashburn, Virginia, United States of America; 3 Genetic Strains Research Center, National Institute of Genetics, Mishima, Japan; 4 Department of Marine Biosciences, Tokyo University of Marine Science and Technology, Tokyo, Japan; 5 Life Science Center for Survival Dynamics, Tsukuba Advanced Research Alliance, University of Tsukuba, Tsukuba, Japan; 6 Faculty of Life and Environmental Sciences, University of Tsukuba, Tsukuba, Japan; 7 PRESTO, Japan Science and Technology Agency, Kawaguchi, Japan; 8 AMED-CREST, Japan Agency for Medical Research and Development, Tokyo, Japan; University of Michigan, United States of America

## Abstract

Stem cell maintenance is established by neighboring niche cells that promote stem cell self-renewal. However, it is poorly understood how stem cell activity is regulated by systemic, tissue-extrinsic signals in response to environmental cues and changes in physiological status. Here, we show that neuropeptide F (NPF) signaling plays an important role in the pathway regulating mating-induced germline stem cell (GSC) proliferation in the fruit fly *Drosophila melanogaster*. NPF expressed in enteroendocrine cells (EECs) of the midgut is released in response to the seminal-fluid protein sex peptide (SP) upon mating. This midgut-derived NPF controls mating-induced GSC proliferation via ovarian NPF receptor (NPFR) activity, which modulates bone morphogenetic protein (BMP) signaling levels in GSCs. Our study provides a molecular mechanism that describes how a gut-derived systemic factor couples stem cell behavior to physiological status, such as mating, through interorgan communication.

## Author summary

Communication between different organs is essential to respond quickly to environmental cues or changes in the physiological status of an organism. Recent studies have shown the existence of humoral factors or hormones, which are transported by the circulatory system to different organs and achieve coordination between them. Here, we have analyzed the communication mechanism between organs that regulates proliferation of germline stem cells (GSCs) in the ovary of the fruit fly *Drosophila melanogaster*. We show that a peptide hormone called neuropeptide F (NPF) is a key player in this process. This peptide is produced in both the brain and the midgut, and, remarkably, we find that only NPF released from the midgut is crucial for controlling post-mating GSC proliferation. Our data suggest that mating stimulates the release of NPF from the endocrine cells of the midgut stimulated by the presence of a seminal peptide. Midgut-derived NPF is then transduced through an NPF-specific G-protein–coupled receptor expressed in the ovary, and this triggers GSC proliferation. Our study identifies an essential interaction between the digestive system and the ovary that regulates the size of stem cell populations in flies depending on mating.

## Introduction

Maintenance and regeneration of adult tissues requires a robust stem cell system that balances self-renewal with differentiation [1]. Because abnormalities in stem cell regulation may result in loss of tissue integrity or tumorigenesis, this robust stem cell system is precisely modulated by local and systemic signals [2]. Stem cells reside in a specialized microenvironment, or niche, where they are exposed to local signals required for stem cell function and identity [3,4]. A number of studies have demonstrated the importance of local niche signals in regulating stem cell identity. Less is known, however, regarding how stem cell activity is regulated by systemic, tissue-extrinsic signals in response to environmental cues and changes in physiological status.

**Fig 1:**
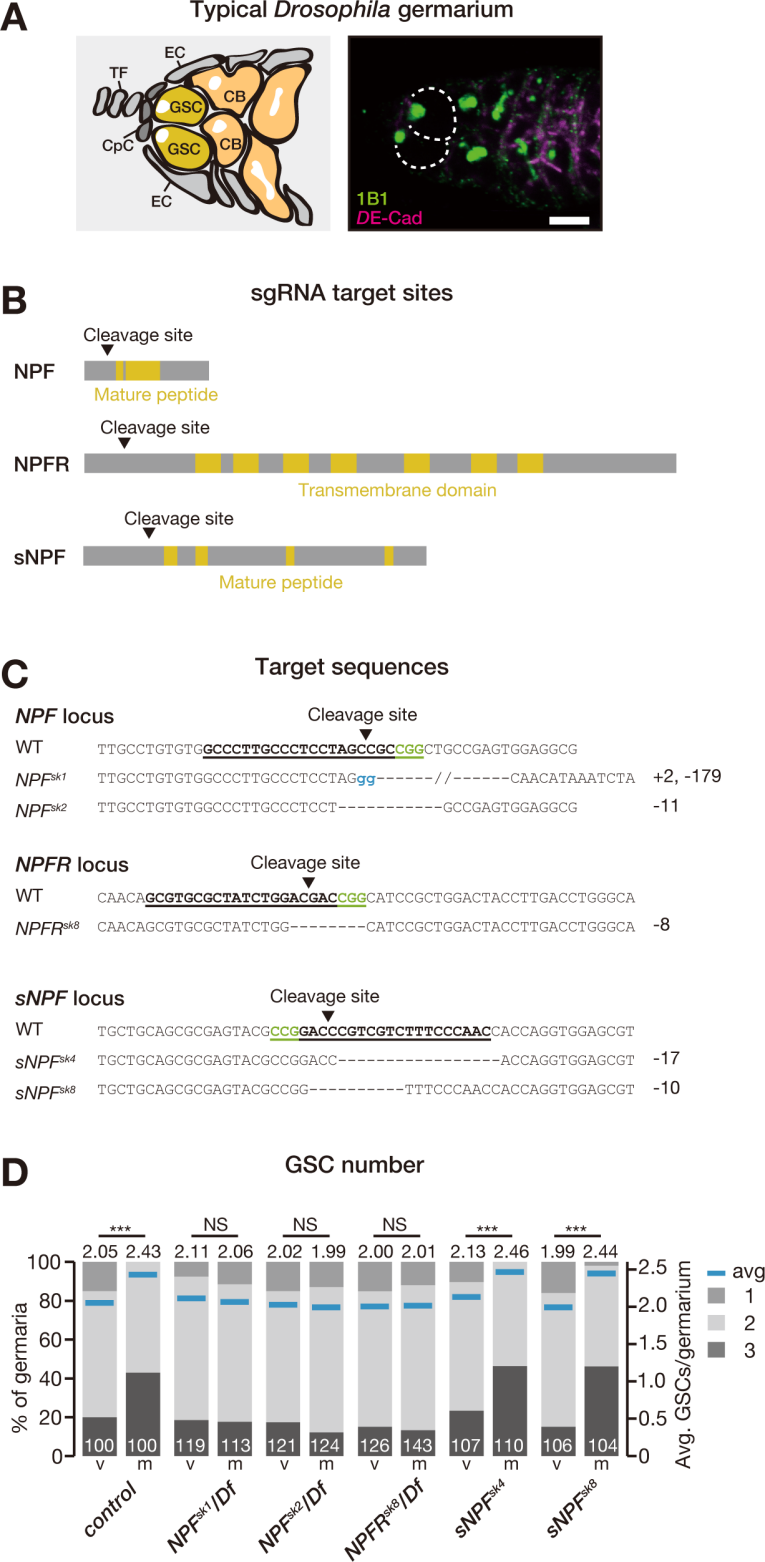
Mating-induced GSC proliferation requires *NPF* and *NPFR*. (A, left) Schematic *Drosophila* germarium. GSCs reside in a niche formed from somatic cells such as CpCs, TF, and ECs. GSCs are identifiable by a stereotypical spectrosome morphology and location (adjacent to CpCs). GSC division produces 1 self-renewing daughter and 1 CB that differentiates into a germline cyst. (A, right) Representative germarium from a WT fly, containing 2 GSCs. Samples were stained with monoclonal antibody 1B1 (green) and anti-DE-cadherin (magenta), which stain the spectrosome and overall cell membranes, respectively. GSCs are indicated by dotted circles. (B) Schematic representation of sgRNA target sites (cleavage sites) in coding sequences of *NPF*, *NPFR*, and *sNPF* genes. Regions encoding the mature NPF and sNPF peptides and the putative transmembrane domains of NPFR are highlighted in yellow. (C) The target locus in each Cas9-induced mutant was PCR-amplified and sequenced. The WT sequence is shown at the top of each set of sequences as a reference. The Cas9-sgRNA target sequence is underlined with the PAM indicated in green. Deleted nucleotides are shown as dashes. Inserted nucleotides are indicated in blue lowercase letters. The indel size is shown next to each sequence. Each of these indel mutations results in a premature stop codon. (D) Frequencies of germaria containing 1, 2, and 3 GSCs (left vertical axis) and the average number of GSCs per germarium (right vertical axis) in virgin (v) and mated (m) female flies. Loss of *NPF* or *NPFR* disrupted the mating-induced increase in GSC number. Flies carrying the CRISPR-induced mutations *NPF*^*SK1*^ or *NPF*^*SK2*^ were crossed with *Df(3R)ED10642*. Mutant flies carrying *NPFR*^*sk8*^ were crossed with *Df(3R)BSC464*. The number of germaria analyzed is indicated inside the bars in D. For statistical analysis, a Wilcoxon rank sum test was used for D. ***P ≤ 0.001. Scale bar = 5 μm in panel A. Underlying data can be found in S1 Data. See also S1 Fig. Cas9, CRISPR-associated protein 9; CB, cystoblast; CpC, cap cell; CRISPR, Clustered Regularly Interspaced Short Palindromic Repeats; EC, escort cell; GSC, germline stem cell; NPF, neuropeptide F; NPFR, neuropeptide F receptor; NS, nonsignificant (*P* > 0.05); sgRNA, single guide RNA; sNPF, short neuropeptide F precursor; TF, terminal filament; WT, wild-type.

The *Drosophila* ovary is one of the most powerful models for studying adult stem cell behavior in vivo [3,5]. This tissue is composed of many chains of developing egg chambers called ovarioles [6]. The most anterior region of each ovariole, the germarium, contains germline stem cells (GSCs) that give rise to the eggs (Fig 1A). GSCs can divide symmetrically to produce a generative cell population or asymmetrically to produce daughter cells called cystoblasts. Each cystoblast undergoes differentiation into 15 nurse cells and 1 oocyte in each egg chamber, which is surrounded by somatic follicle cells. Therefore, the balance between self-renewal and differentiation of GSCs plays a pivotal role in regulating oogenesis because disruption of this balance may cause germ cell depletion, infertility, or tumorigenesis [1].

*Drosophila* female GSCs are anchored to the somatic niche, which comprises cap cells, escort cells, and terminal filaments. The niche produces local signals such as the bone morphogenetic protein (BMP) ligand Decapentaplegic (Dpp), which activates its receptors Saxophone (Sax), Punt (Put), and Thickveins (Tkv) expressed in GSCs to induce GSC division and maintenance [7]. Local signals from the niche have a crucial role in regulating reproduction because impairment of niche signals can cause germ cell depletion, infertility, and tumorigenesis. On the other hand, it is well known that animal reproduction is also coordinated by signals from the external environment [8,9]. One such example involves nutrients, which are important materials for producing eggs. On a protein-poor diet, egg production is restricted through blocking vitellogenesis [8]. A protein-poor diet also results in a reduction of GSC division, which is mediated by neural-derived *Drosophila* insulin-like peptides (DILPs) [10]. Moreover, in response to nutrients, GSC maintenance is controlled by the adipocyte metabolic pathway [11,12]. Another example of an environmental cue that affects reproduction is mating. Mated females show a dramatic increase in egg production, which is induced by a male-derived peptide from seminal fluid termed sex peptide (SP) [13]. We have previously reported that neural SP signaling also promotes GSC proliferation through its effects on the biosynthesis of ecdysteroids (insect steroid hormones) in the ovary [14,15]. Taken together, these findings suggest that GSC proliferation and maintenance are modulated by tissue-extrinsic signals in response to environmental cues.

Here, we present a series of new findings that reveal a novel and fundamental interorgan communication mechanism controlling GSC proliferation in response to mating. We demonstrate that *Drosophila* neuropeptide F (NPF), a homolog of mammalian neuropeptide Y (NPY), acts as a key regulator of mating-induced GSC proliferation in *Drosophila* females. Although *NPF* is expressed in both the brain and the midgut, we found that only the enteroendocrine-derived peptide—not neuronal NPF—is required for activation of GSCs after mating. The NPF protein is highly accumulated in enteroendocrine cells (EECs) of the middle midgut of virgin female flies and is released in response to SP-dependent signaling upon mating. Through fly injection and ex vivo ovary cultures with synthetic peptide, we show that NPF signaling is sufficient for increasing GSC number in virgin female flies. Notably, after mating, midgut-derived NPF acting on the ovaries through the NPF receptor (NPFR) up-regulates BMP signaling levels in GSCs to induce their proliferation. Our findings describe a mechanism of gut-to-ovary communication that couples stem cell behavior to physiological status by sensing external cues such as mating. Considering NPY’s role in regulating reproduction in many animal species, our study also provides new insights into the role of interorgan communication during animal germline development.

## Results

### Disruption of NPF function in midgut EECs impairs mating-induced GSC proliferation

We employed a genetic screen using *Drosophila* fly lines carrying Clustered Regularly Interspaced Short Palindromic Repeats (CRISPR)/CRISPR-associated protein 9 (Cas9)-generated mutations in neuropeptide-encoding genes [16] and identified stem cell phenotypes in mutants of NPF (Fig 1B and 1C). In control flies, mated female flies had more GSCs than virgin female flies (Fig 1D), as we reported previously [14]. In contrast, the mating-induced increase in GSC number was suppressed in genetic null mutants for *NPF* itself or for the gene encoding the NPFR (Fig 1B–1D). We also found that genetic null mutant flies of short neuropeptide F precursor (sNPF), encoding an RxRFamide neuropeptide related to NPF, showed a normal increase in GSC number after mating (Fig 1D), suggesting that the GSC-suppression phenotype is specific to NPF signaling.

Our immunostaining analysis confirmed the presence of anti-NPF signals in the brain [17,18] and EECs of the middle midgut [19,20] in control flies, but not in *NPF* mutants (S1A Fig). A number of previous studies have already reported that neuronal NPF regulates multiple aspects of physiology and behavior in adult flies, such as circadian rhythm, alcohol sensitivity, male courtship behavior, and food intake [17,18,21–24], whereas the function of EEC-derived NPF remains unclear [20,25]. We first investigated whether neuronal NPF function is required for the normal increase in GSC number induced by mating. However, although RNA interference (RNAi)-mediated knockdown of *NPF* either pan-neuronally (using *nSyb-GAL4*) or in neuroendocrine cells (using *386Y-GAL4*) resulted in a drastic decrease in NPF protein levels in the brain (S1B Fig), neither manipulation had any effect on post-mating GSC number (S1C Fig). We also confirmed that RNAi driven by neither the *nSyb-GAL4* nor *386Y-GAL4* driver resulted in reduced NPF levels in midgut EECs (S1B Fig).

**Fig 2:**
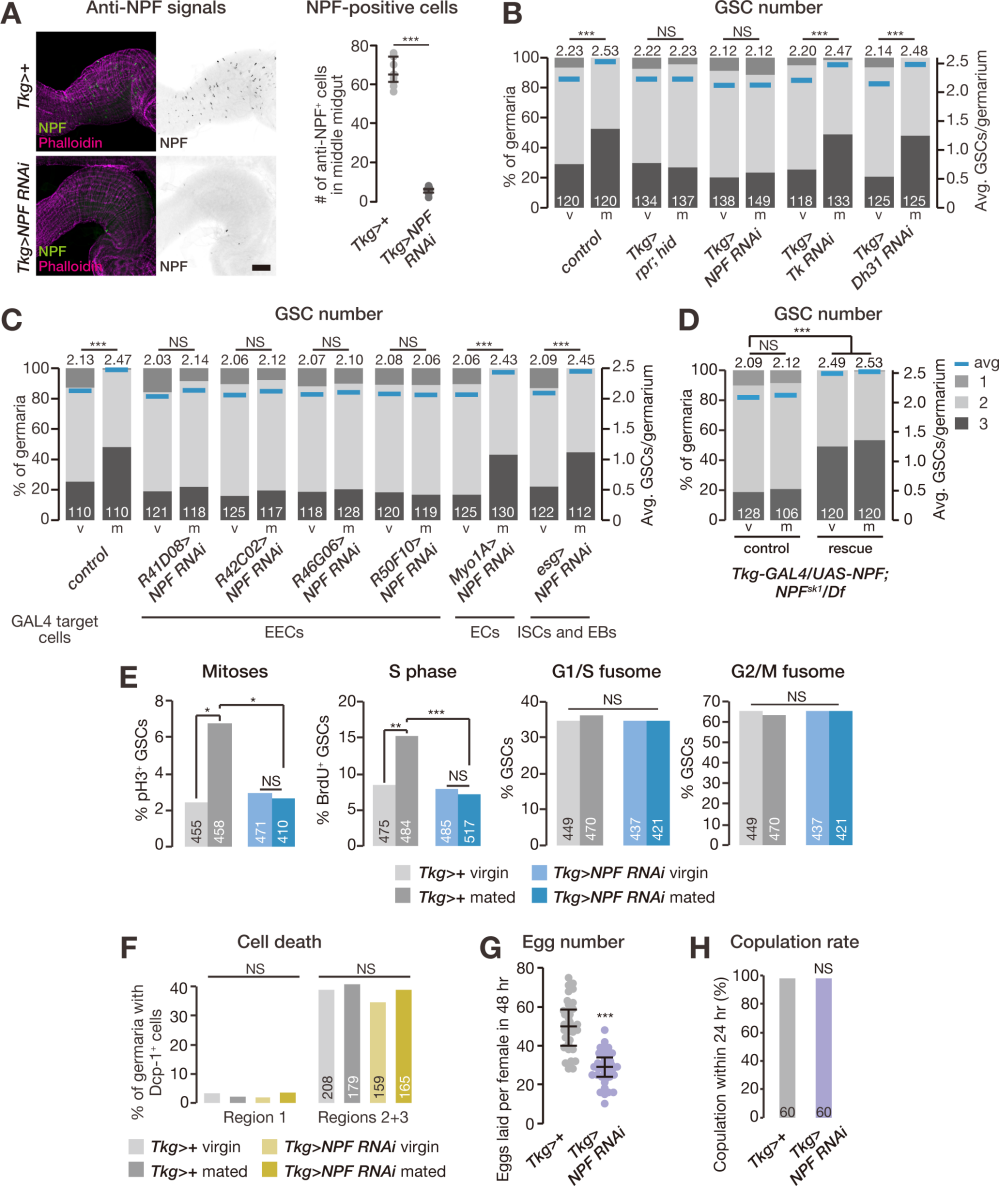
Midgut-derived NPF promotes mating-induced GSC proliferation. (A) The number of NPF-positive cells was dramatically reduced in the middle midgut of *Tkg-GAL4*>*NPF*^*RNAi*^ animals. (B–D) Frequency of germaria containing 1, 2, and 3 GSCs (left axis) and the average number of GSCs per germarium (right axis) in virgin (v) and mated (m) female flies. (B) Removal of *Tkg-GAL4*-positive cells by induced apoptosis or *NPF* RNAi driven by *Tkg-GAL4* suppressed the increase in GSC number after mating. RNAi against *Tk* or *Dh31*, which are also expressed in these cells, driven by *Tkg-GAL4* had no effect on mating-induced increase in GSC number. (C) *NPF* RNAi driven by several *GAL4* drivers targeting EECs disrupted the increase in GSC number after mating, whereas *NPF* RNAi driven by esg-*GAL4* (ISCs and EBs) or *Myo1A*-*GAL4* (ECs) had no effect on the mating-induced increase in GSC number. (D) The post-mating GSC proliferation defect observed in *NPF* genetic null mutants was rescued by overexpression of the NPF transgene under the control of *Tkg-GAL4*. Rescue, *Tkg-GAL4/UAS-NPF; NPF*^*sk1*^*/Df(3R)ED10642*. Control, *Tkg-GAL4/+; NPF*^*sk1*^*/Df(3R)ED10642* or *UAS-NPF/+; NPF*^*sk1*^*/Df(3R)ED10642*. (E) Mating increased the fraction of GSCs in mitosis (measured by pH3 labeling) and S phase (measured by BrdU incorporation), but not G1/S or G2/M fusome morphologies. The cell cycle promotion observed in GSCs after mating was suppressed in *Tkg-GAL4*>*NPF*^*RNAi*^ animals. (F) Percentages of germaria containing cleaved Dcp-1-positive cells in either region 1 (anterior-most region; containing GSCs, cystoblasts, and dividing cysts) or regions 2 and 3 (containing 16-cell cysts and follicle cells) did not change after mating or *NPF* RNAi. (G) The total number of eggs produced by females within 48 hours was reduced in *Tkg-GAL4*>*NPF*^*RNAi*^ animals. (H) Receptivity of virgin females was scored as the percentage of females that copulated within 24 hours. The number of germaria analyzed is shown inside the bars in B–F and H. Dots represent the relative intensity of NPF in a single middle midgut (panel A) or the number of eggs produced by a single female fly (panel G); lines represent the median, and whiskers represent the interquartile range. For statistical analysis, a Wilcoxon rank sum test was used for panel B–D. Student *t* test with Holm’s correction was used for panel A and G. Fisher’s exact test with Holm’s correction was used for panel E, F, and H. ***P ≤ 0.001, **P ≤ 0.01, and *P ≤ 0.05; NS, nonsignificant (*P* > 0.05). Scale bar = 50 μm in panel A. Underlying data can be found in S1 Data. See also S2, S3 and S4 Figs. BrdU, bromodeoxyuridine; Dcp-1, Death caspase-1; Dh31, diuretic hormone 31; EB, enteroblast; EC, enterocyte; EEC, enteroendocrine cell; GSC, germline stem cell; hid, head involution defective; ISC, intestinal stem cell; NPF, neuropeptide F; pH3, phospho-histone H3; RNAi, RNA interference; rpr, reaper; Tk, Tachykinin; Tkg-GAL4, Tk-gut-GAL4.

Therefore, we next examined whether the mating-induced increase in GSC number is controlled by midgut-expressed *NPF*, the other potential source of NPF protein. For this purpose, we utilized the *Tk-gut-GAL4* (*Tkg-GAL4*) driver because this *GAL4* driver is known to be active in a restricted population of midgut cells, including *NPF*-positive EECs [20], but not in the ovary (S2A Fig). Immunostaining analysis with anti-NPF antibody revealed that *Tkg-GAL4*-mediated transgenic RNAi against *NPF* (hereafter *Tkg>NPF*^*RNAi*^) dramatically reduced the number of NPF-positive cells in the middle midgut compared with controls (Fig 2A). It should be noted that *Tkg-GAL4* is also active in some neuronal cells in the brain and the ventral nerve cord (VNC; S2A and S2B Fig); however, *Tkg>NPF*^*RNAi*^ animals did not show a significant reduction in NPF levels in these neuronal cells (S2C Fig). In *Tkg>NPF*^*RNAi*^ females, we found that the mating-induced increase in GSC number was severely impaired (Fig 2B). In addition, we performed RNAi-mediated knockdown of *NPF* driven by several other midgut-*GAL4* drivers. Suppression of the increase in GSC number after mating was observed with 4 of these drivers (Fig 2C), which are active in middle midgut EECs [26] but not in ovaries or NPF-positive neurons (S3 Fig). On the other hand, *NPF* RNAi in enterocytes (using *Myo1A-GAL4*) or intestinal stem cells and enteroblasts (*esg-GAL4*) had no effect on the mating-induced increase in GSC number (Fig 2C). We found that the GSC proliferation defect in *NPF* genetic null mutants was rescued by overexpression of the *NPF* transgene under the control of the *Tkg-GAL4* driver (Fig 2D).

*Tkg-GAL4*-positive cells also express other gut peptide hormone genes, including *Tachykinin* (*Tk*) and *diuretic hormone 31* (*Dh31*) [20]. However, transgenic RNAi against either *Tk* or *Dh31* driven by *Tkg-GAL4* had no effect on post-mating increase in GSC number (Fig 2B). On the other hand, ablating the *Tkg-GAL4*-positive cells by expressing the cell death–inducing factor *reaper* (*rpr*) and *head involution defective* (*hid*) led to suppression of the increase in GSC number after mating (Fig 2B). Taken together, these results suggest that EECs play an important role in regulating the mating-induced increase in GSC number, mainly through the function of NPF.

We next examined whether midgut-derived NPF controls GSC division. For this purpose, we counted the number of GSCs in M phase and S phase by staining with anti-phospho-histone H3 (pH3) and bromodeoxyuridine (BrdU), respectively, in control and *Tkg>NPF*^*RNAi*^ adult females. In control female flies, we found that mating increased the frequency of GSCs in both M and S phases (Fig 2E). We also monitored GSC fusome morphology as an indicator of cell cycle phase [27] and did not observe any difference in the frequency of GSCs in G2/M and G1/S phases (Fig 2E). In *Tkg>NPF*^*RNAi*^ animals, the increase in the fraction of GSCs in M and S phases was suppressed (Fig 2E), suggesting that midgut-derived NPF promotes GSC progression through both DNA replication and mitosis. We also monitored the fraction of apoptotic cells in the germarium by staining with anti-cleaved Death caspase-1 (Dcp-1), a marker for apoptotic cells [28]. The number of apoptotic cells did not change in *Tkg>NPF*^*RNAi*^ female flies compared with controls (Fig 2F), suggesting that the lack of post-mating GSC-number increase seen with *NPF* RNAi was not caused by increased cell death but mainly by a lack of NPF-induced cell proliferation.

Consistent with their GSC proliferation phenotype, we found that mated *Tkg>NPF*^*RNAi*^ female flies laid fewer eggs than mated control females (Fig 2G). Taken together, these data indicate that midgut-derived NPF has a positive impact not only on GSC proliferation but on reproductive fitness after mating as well. To rule out the possibility that the GSC phenotype was due to the absence of mating, we confirmed that the copulation rate of *Tkg>NPF*^*RNAi*^ female flies did not change compared with that of control female flies by using males expressing *GFP* in their sperm (Fig 2H). These findings are all consistent with the idea that NPF from EECs modulates GSC division after mating.

### Midgut-derived NPF does not affect gut remodeling after mating

In *Drosophila* females, mating induces midgut epithelium remodeling, including increases in gut size and in the number of mitotic cells, which is essential for enhancing reproductive output [29,30]. Thus, there may be a possibility that the suppression of mating-induced GSC proliferation in *Tkg>NPF*^*RNAi*^ animals is indirectly caused by the dysfunction of midgut remodeling after mating. We therefore examined whether midgut-derived NPF affects gut size or the number of mitotic cells in the midgut after mating; however, *Tkg>NPF*^*RNAi*^ females displayed normal increases in mitosis in the midgut epithelium and posterior midgut diameter (S4A and S4B Fig). This suggests that midgut NPF is not involved in tissue remodeling after mating and that the GSC phenotypes in *Tkg>NPF*^*RNAi*^ female flies are not due to an indirect effect of defective mating-induced remodeling in the midgut epithelium.

### Mating affects NPF accumulation in EECs

**Fig 3:**
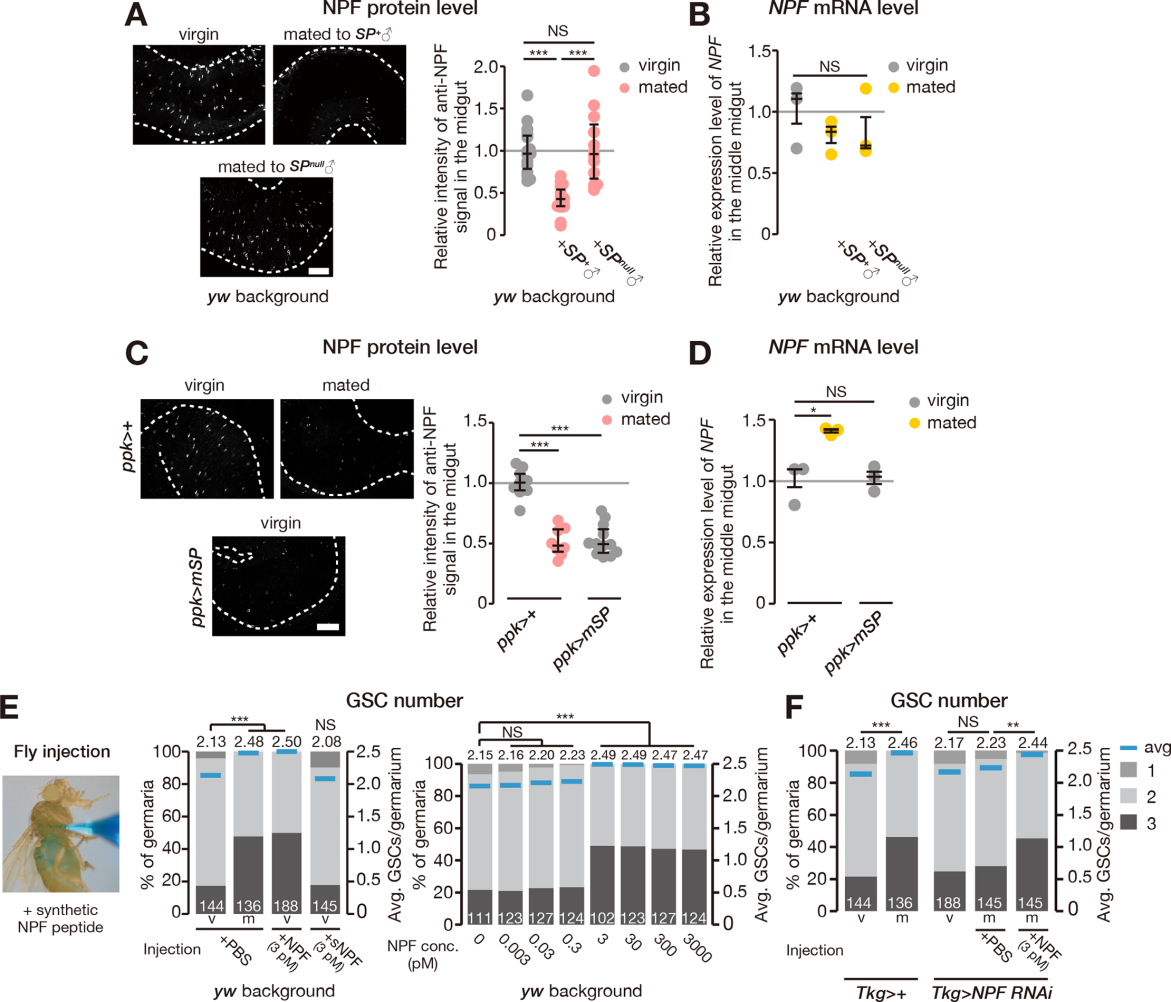
Mating induces NPF accumulation in EECs, and exogenous NPF increases GSC. (A–D) Levels of NPF protein and *NPF* transcript in the middle midgut. (A, C) Representative images of anti-NPF immunostaining in the middle midgut are shown on the left. Quantification of anti-NPF signal intensity in the middle midgut is shown on the right graph. (A) Anti-NPF signal accumulated in virgin female flies and females mated with *SP*-null mutant males (*SP*^0^/*SP*^*Δ130*^). (B) Transcript abundance of *NPF* in the middle midgut did not change after mating with wild-type or *SP*-null male flies. (C) NPF release was induced without mating by overexpressing *mSP* in *ppk*-positive neurons (*ppk-GAL4>mSP*). (D) Transcript abundance of *NPF* in the middle midgut was not changed by this manipulation. (E, F) Fraction of germaria containing 1, 2, and 3 GSCs (left axis) and the average number of GSCs per germarium (right axis) in virgin (v) and mated (m) female flies. (E) Microinjection of synthetic NPF peptide, but not sNPF (Bm-sNPF), into virgin female flies induced an increase in GSC number. NPF-dependent increase in GSC number occurred after injecting 3–3,000 pM (but not 0–0.3 pM) NPF. (F) The GSC-number phenotype of *NPF* RNAi animals was rescued by injecting synthetic NPF peptide. Dots represent the relative signal intensity of anti-NPF in single middle midguts (panel A and C) or the relative expression levels of *NPF* in the middle midgut (panel B and D); lines represent the median, and whiskers represent the interquartile range. The number of germaria analyzed is shown inside the bars in panel E and F. For statistical analysis, a Wilcoxon rank sum test with Holm’s correction was used for panel A, C, E, and F. Student *t* test with Holm’s correction was used for panel B and D. ***P ≤ 0.001, **P ≤ 0.01, and *P ≤ 0.05; NS, nonsignificant (*P* > 0.05). Scale bar = 50 μm in panel A and C. Underlying data can be found in S1 Data. See also S5 Fig. GSC, germline stem cell; mSP, membrane-tethered SP; NPF, neuropeptide F; ppk, pickpocket; RNAi, RNA interfererence; sNPF, short neuropeptide F precursor; SP, sex peptide; Tkg-GAL4, Tk-gut-GAL4.

To investigate the relationship between mating and NPF in EECs, we performed immunostaining with anti-NPF antibody on the midguts of virgin and mated female flies. Anti-NPF signal was stronger in the EECs of virgin females compared with mated females (Fig 3A). In contrast, mating did not alter *NPF* mRNA abundance in the middle midgut (Fig 3B), indicating that the observed change in NPF protein levels was not due to transcriptional regulation. This situation was reminiscent of the case of *Drosophila* insulin-like peptide 2 (Dilp2) because it is well known that increased Dilp2 protein level in insulin-producing cells reflects decreased Dilp2 release into the hemolymph when *dilp2* transcription is constant [31]. Similarly, although immunostaining alone cannot completely rule out the contribution of post-transcriptional regulation of NPF, these results do imply that mating promotes NPF release from EECs.

### SP signaling promotes NPF release from middle midgut EECs upon mating

Our previous study [14] revealed that mating-induced GSC proliferation is mediated by the male seminal-fluid component SP, which plays a central role in triggering dramatic changes in female physiology and behavior after mating [13,32]. SP is received by female neurons expressing the sex peptide receptor (SPR), resulting in the silencing of these neurons [33,34]. We found that female flies mated with male flies lacking *SP* showed NPF accumulation in middle midgut EECs without significant changes in *NPF* mRNA levels (Fig 3A and 3B), suggesting that male-derived SP regulates NPF release from EECs upon mating.

The silencing of *SPR*-positive neurons located on the oviduct, which also express pickpocket (ppk) [35], is particularly important for inducing female GSC proliferation after mating [14]. We found that the expression of a transgene encoding membrane-tethered SP (mSP) in *ppk*-positive female neurons decreased NPF protein levels in EECs, even in virgin female flies (Fig 3C), while *NPF* mRNA levels in the middle midgut were not significantly altered (Fig 3D). In contrast, NPF protein levels did not change after expression of *mSP* with the *Tkg-GAL4* driver (S5A and S5B Fig). These results suggest that neuronal—but not midgut—SP signaling is both necessary and sufficient for post-mating NPF release from EECs.

We also examined whether neuronal inactivation of *SPR*-positive neurons was sufficient to reduce NPF accumulation in EECs in virgin female flies, as the binding of SP to SPR silences *SPR*-positive neurons [36]. We utilized a mutant of shibire (shi^ts1^) that blocks synaptic vesicle release in a temperature-dependent manner [37]. Normal NPF accumulation was observed at the permissive temperature in virgin female flies overexpressing *shi*^*ts1*^ (S5C Fig). On the other hand, when *SPR*-positive neurons were silenced in virgin females at the restrictive temperature to mimic mating, NPF protein levels in middle midgut EECs were reduced without any significant changes in *NPF* mRNA levels (S5C and S5D Fig). These results suggest that NPF release from EECs is induced by silencing the transmitter-release activity of some neurons within the *SPR*-positive population.

To further examine the necessity and sufficiency of circulating NPF in inducing the increase in GSC number, we manually delivered synthetic NPF peptide by injecting it using glass needles into adult females. NPF injection into wild-type virgin females resulted in a significant increase in GSC number compared with injection of phosphate-buffered saline (PBS) vehicle (Fig 3E). No such increase in GSC number was observed after injection of synthetic sNPF peptide (Fig 3E). Moreover, we observed that the impairment in GSC increase seen in mated *Tkg>NPF*^*RNAi*^ animals was restored by NPF injection but not by control PBS injection (Fig 3F). These results suggest that elevation in circulating NPF levels is both necessary and sufficient to trigger mating-induced GSC proliferation.

### NPF induces an increase in GSC number by acting on the ovary

**Fig 4:**
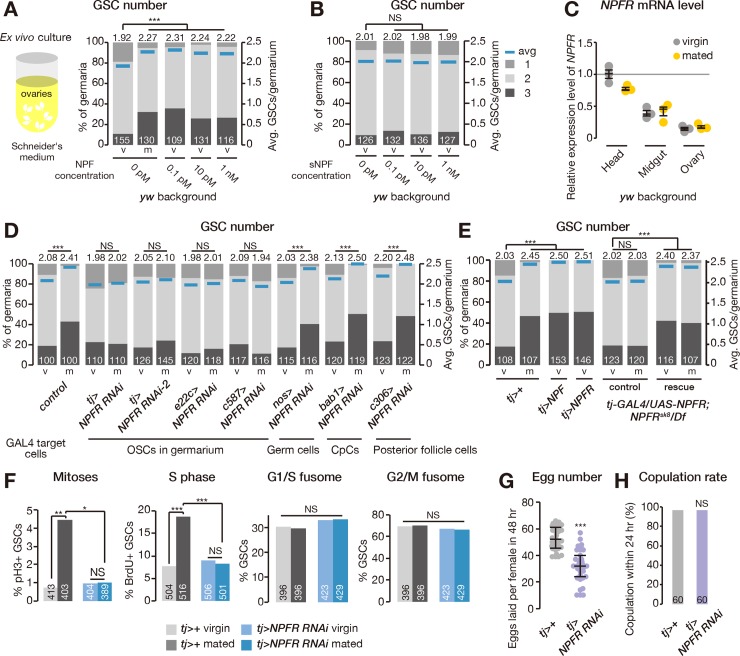
Ovarian NPFR controls mating-induced GSC proliferation. (A, B, D, E) Frequency of germaria containing 1, 2, and 3 GSCs (left axis) and the average number of GSCs per germarium (right axis) in virgin (v) and mated (m) female flies. The addition of synthetic NPF peptide (panel A), but not of sNPF (panel B), to the ex vivo ovary culture medium was sufficient to increase GSC number. (C) *NPFR* transcripts were detected in the ovary, although expression levels were much lower than in the head and midgut. (D) *NPFR* RNAi driven by *tj-GAL4*, *e22c-GAL4*, or *c587-GAL4* (targeting germarium somatic cells) inhibited the mating-induced increase in GSC number. The increase in GSC number after mating was not altered by *NPFR* RNAi driven by *nos-GAL4*, *bab1-GAL4*, or *c306-GAL4* (germ, cap, and posterior follicle cells, respectively). (E) Overexpressing *NPF* or *NPFR* in the somatic cells of the germaria was sufficient to increase GSC number in virgin female flies. The GSC proliferation defect in *NPFR* genetic null mutants was rescued by overexpressing the *NPFR* transgene under the control of *tj-GAL4*. Rescue, *tj-GAL4/UAS-NPFR; NPFR*^*sk8*^*/Df(3R)BSC464*. Control, *tj-GAL4/+; NPFR*^*sk8*^*/Df(3R)BSC464* or *UAS-NPFR/+; NPFR*^*sk8*^*/Df(3R)BSC464*. (F) Mating increased the frequency of GSCs in M (measured by pH3 labeling) and S (measured by BrdU incorporation) phases, but not G1/S or G2/M fusome morphologies. Cell cycle promotion in GSCs after mating was suppressed in *tj-GAL4*>*NPFR*^*RNAi*^ animals. (G) The total number of eggs produced by females within 48 hours was reduced in *tj-GAL4*>*NPFR*^*RNAi*^ animals. (H) Receptivity of virgin females was scored as the percentage of females that copulated within 24 hours. The number of germaria analyzed is shown inside the bars in panel A, B, D–F, and H. For statistical analysis, a Wilcoxon rank sum test with Holm’s correction was used for A, B, D, and E. Fisher’s exact test with Holm’s correction was used for panel F and H. Student *t* test was used for panel G. ***P ≤ 0.001, **P ≤ 0.01, and *P ≤ 0.05; NS, nonsignificant (*P* > 0.05). Underlying data can be found in S1 Data. See also S6 Fig. BrdU, bromodeoxyuridine; CpC, cap cell; GSC, germline stem cell; NPF, neuropeptide F; NPFR, neuropeptide F receptor; OSC, ovarian somatic cell; pH3, phospho-histone H3; RNAi, RNA interference; sNPF, short neuropeptide F precursor.

The next question to be addressed is whether the NPF signal is directly received by the ovary or is transmitted via other tissues to control mating-induced GSC proliferation. We therefore performed ex vivo ovary culture experiments with synthetic NPF peptide. In this experiment, we dissected ovaries from virgin female flies and cultured them in Schneider’s *Drosophila* cell culture medium with or without synthetic NPF peptide for 1 day. We found that dissected virgin ovaries cultured with the NPF peptide possessed more GSCs than controls (Fig 4A), indicating that NPF directly affects the ovary in controlling GSC number. This increase was not observed when the ovaries were cultured with synthetic sNPF peptide (Fig 4B), suggesting that the observed response is specific to NPF.

### NPFR activity in the ovarian somatic cells controls mating-induced GSC proliferation

To further understand the role of NPF signaling in the ovary, we focused on the NPFR (CG1147) in *Drosophila* [38]. We confirmed that *NPFR* is expressed in the ovary (Fig 4C); however, expression levels in this tissue were much lower than in the head or midgut, the tissues previously reported to express *NPFR* [17,38]. We also found that the increase in GSC number after mating was suppressed by transgenic *NPFR* RNAi driven by *tj-GAL4*, *e22c-GAL4*, or *c587-GAL4* drivers (Fig 4D), which are known to be active in the somatic cells of ovarian germaria, including escort and follicle cells [39–42]. Conversely, we found that overexpression of either *NPFR* or *NPF* driven by *tj-GAL4* was sufficient to increase GSC number in virgin females (Fig 4E). On the other hand, no suppression of the increase in GSC number was observed when we knocked down *NPFR* function in germ cells (using *nos-GAL4*), cap cells (*bab1-GAL4*), or posterior follicle cells (*c306-GAL4*; Fig 4D). Although both *tj-GAL4* and *c587-GAL4* drive expression in the brain and VNC (S6A Fig), pan-neuronal RNAi knockdown of *NPFR* function did not affect the mating-induced increase in GSC number (S6B Fig). We also confirmed that intestinal RNAi knockdown of *NPFR* function did not disrupt the mating-induced increase in GSC number (S6C Fig). We found that the GSC proliferation defect in *NPFR* genetic null mutants was rescued by overexpression of the *NPFR* transgene under the control of *tj-GAL4* (Fig 4E).

We next examined cell cycle progression of GSCs in ovarian *NPFR*-knockdown females. This manipulation led to phenotypes similar to those seen in *Tkg>NPF*^*RNAi*^ female flies—namely, a decrease in GSC frequency in M and S phases after mating without any effects on G1/S or G2/M phase transitions (Fig 4F).

Similar to the case of *Tkg>NPF*^*RNAi*^, mated *tj>NPFR*^*RNAi*^ female flies laid fewer eggs than mated control females (Fig 4G). We also found that the copulation rate of *tj>NPFR*^*RNAi*^ animals did not change in comparison with control female flies by using males expressing *GFP* in their sperm (Fig 4H), ruling out the possibility that the GSC phenotype was due to the absence of mating. Taken together, these data suggest that *NPFR* in ovarian somatic cells is necessary and sufficient for positively controlling mating-induced GSC proliferation and reproductive fitness.

### NPF-dependent induction of the increase in GSC number requires NPFR in the ovary

**Fig 5:**
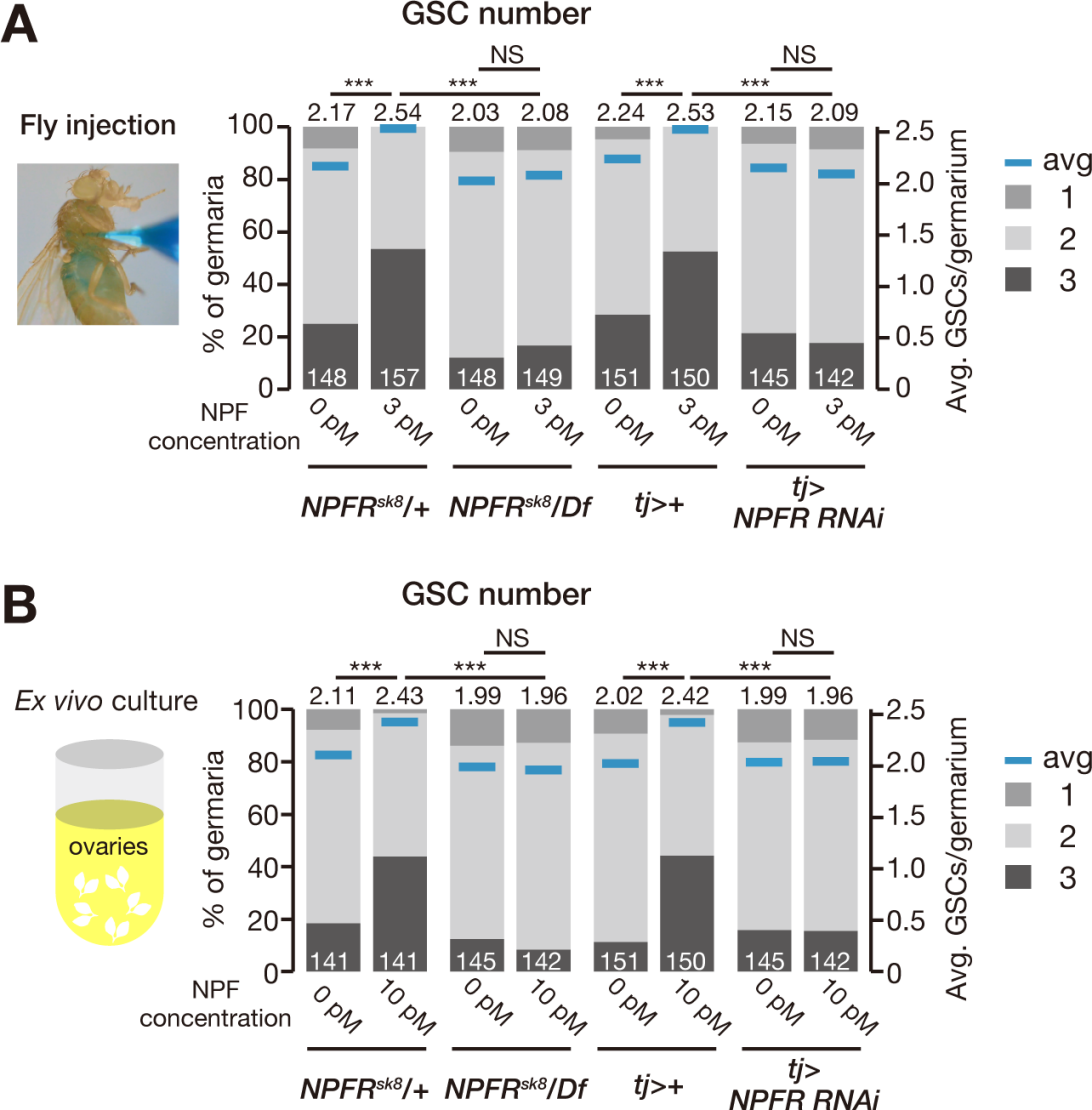
NPF-dependent induction of GSC increase requires NPFR in the ovary. (A, B) Frequency of germaria containing 1, 2, and 3 GSCs (left axis) and the average number of GSCs per germarium (right axis) in virgin (v) and mated (m) female flies. Loss of *NPFR* (*NPFR*^*sk8*^/*D(3R)BSC464*) or ovarian knockdown of *NPFR* (*tj-GAL4*>*NPFR*^*RNAi*^) blocked the NPF-induced increase in GSC number after fly injection (panel A) or ex vivo ovary cultures (panel B). The number of germaria analyzed is shown inside the bars. For statistical analysis, a Wilcoxon rank sum test with Holm’s correction was used. ***P ≤ 0.001. Underlying data can be found in S1 Data. GSC, germline stem cell; NPF, neuropeptide F; NPFR, neuropeptide F receptor; NS, nonsignificant (*P* > 0.05); RNAi, RNA interference.

Remarkably, we found that the increase in GSC number induced by in vivo injection or ex vivo ovary culture with synthetic NPF peptide was completely suppressed in *NPFR*-knockdown animals (Fig 5A and 5B), suggesting that *NPFR* expressed in the ovary is epistatic to *NPF* for controlling GSC proliferation. Because the midgut and ovaries are distinct and separate organs, midgut-derived NPF must remotely act on the ovary to control GSC proliferation in response to mating. Therefore, based on our data described above, we hypothesized that a mating stimulus triggers the release of NPF from EECs into the hemolymph, after which the circulating NPF signal can be received by the ovary.

### NPF signaling regulates Dpp signaling in GSCs of mated female flies

**Fig 6:**
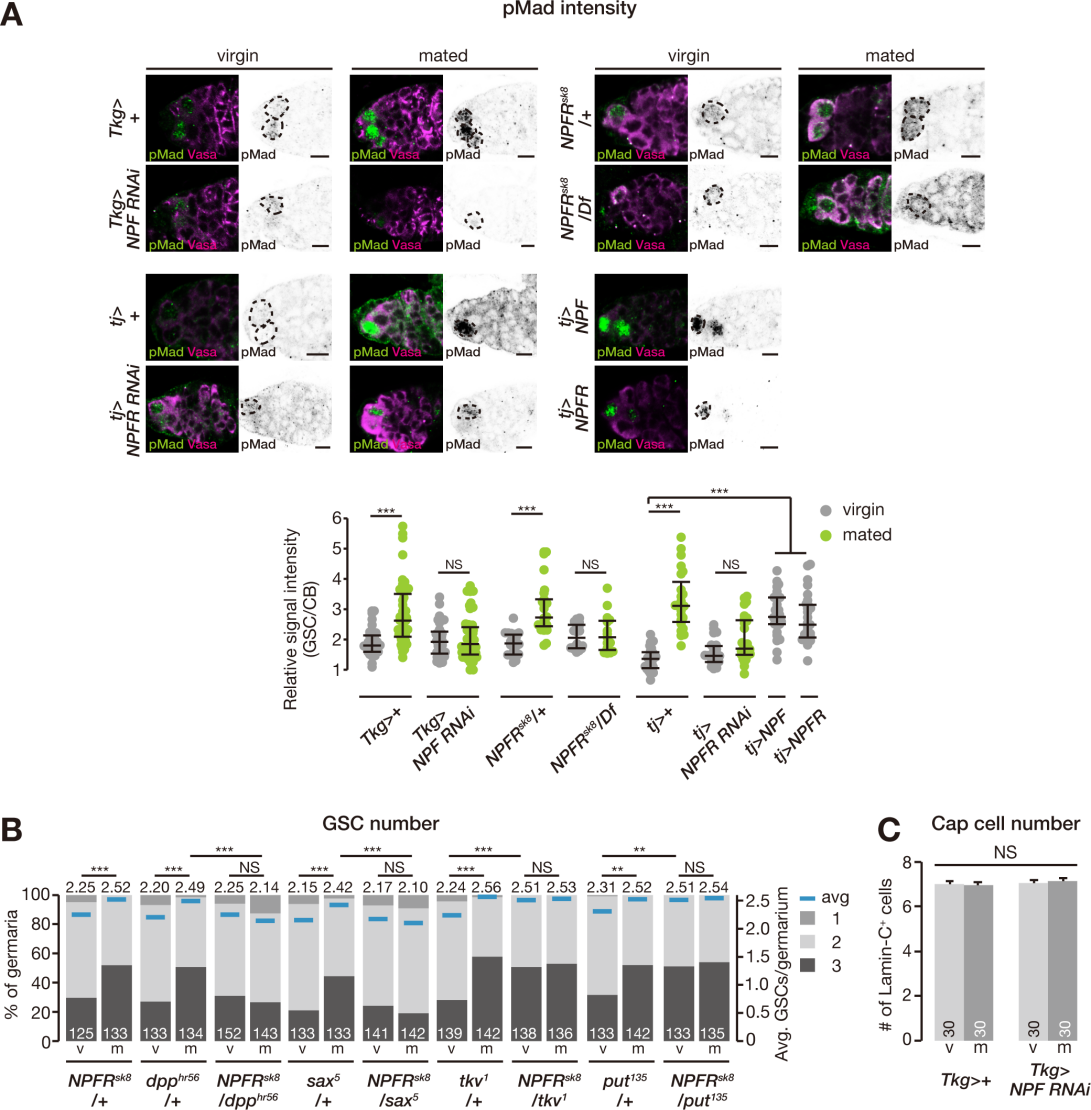
NPF signaling regulates BMP signaling levels in GSCs and promotes GSC self-renewal towards symmetric GSC divisions. (A) Representative images of adult female germaria immunostained with anti-pMad antibody (green) and anti-Vasa antibody (germ cell marker; magenta) are shown at the top (GSC, dotted lines). Quantification of relative pMad intensity in GSCs, which was normalized to pMad intensity in CBs, is shown in the bottom graph. For quantification of pMad intensity, the cell boundaries of GSCs and CBs were determined by anti-Vasa staining. Mating-induced increase in pMad expression was suppressed by intestinal *NPF* RNAi (*Tkg-GAL4*>*NPF*^*RNAi*^), ovarian *NPFR* (*tj-GAL4>NPFR*^*RNAi*^), or loss of *NPFR* function (*NPFR*^*sk8*^/*Df(3R)BSC464*). Increase in pMad signal intensity in GSCs was induced by ovarian overexpression of *NPF* or *NPFR*. (B) Frequency of germaria containing 1, 2, and 3 GSCs (left axis) and the average number of GSCs per germarium (right axis) in virgin (v) and mated (m) female flies. The mating-induced increase in GSC number was disrupted in the double heterozygous mutant for *NPFR* and BMP signaling (*NPFR*^*sk8*^*/dpp*^*hr56*^ or *NPFR*^*sk8*^/*sax*^*5*^). (C) The number of cap cells did not change after mating or *NPF* RNAi driven by *Tkg-GAL4*. Each dot represents the relative intensity of pMad in a single germarium, with lines representing the median and whiskers representing the interquartile range in panel A. The number of germaria analyzed is shown inside the bars in panel B and C. Values are presented as the mean with standard error of the mean in panel C. For statistical analysis, a Wilcoxon rank sum test with Holm’s correction was used for panel A and B. Student *t* test with Holm’s correction was used for panel C. ***P ≤ 0.001 and **P ≤ 0.01; NS, nonsignificant (*P* > 0.05). Scale bar = 5 μm. Underlying data can be found in S1 Data. See also S7 Fig. BMP, bone morphogenetic protein; CB, cystoblast; Dpp, Decapentaplegic; GSC, germline stem cell; NPF, neuropeptide F; NPFR, neuropeptide F receptor; pMad, phosphorylated Mad; Put, Punt; Sax, Saxophone; RNAi, RNA interference; *Tkg-GAL4*, *Tk-gut-GAL4*; Tkv, Thickveins.

GSC maintenance and proliferation are controlled by signals from the GSC niche, in particular Dpp—the fly counterpart to BMPs [1]. We therefore examined whether down-regulation of NPF signaling affects Dpp signaling by measuring the level of phosphorylated Mad (pMad), a readout of Dpp signaling activation in cells including GSCs [43]. We found that mating increased pMad levels in GSCs of control flies (Fig 6A). On the other hand, *Tkg>NPF*^*RNAi*^ led to a reduction in pMad levels in GSCs of mated female flies (Fig 6A). The same phenotype was also observed in ovarian *NPFR* RNAi female flies and genetic null alleles of *NPFR* (Fig 6A). Conversely, overexpression of *NPF* or *NPFR* in ovarian somatic cells resulted in elevated pMad levels in the GSCs of virgin female flies (Fig 6A).

We also tested genetic interactions between NPF and Dpp signaling pathways in controlling the mating-induced increase in GSC number. We counted GSCs in double heterozygous mutant flies carrying *NPFR*^*sk8*^ and one of the Dpp pathway mutations, *dpp*^*hr56*^, *sax*^*5*^, *tkv*^*1*^, or *put*^*135*^. The mating-induced increase in GSC number was disrupted in the double heterozygous mutant flies carrying *NPFR*^*sk8*^/*dpp*^*hr56*^ and *NPFR*^*sk8*^/*sax*^*5*^ (Fig 6B). On the other hand, the opposite phenotype of increased GSC number in virgin females was observed in *NPFR*^*sk8*^/*tkv*^*1*^ and *NPFR*^*sk8*^/*put*^*135*^ flies (Fig 6B; see Discussion). These results suggest that midgut-derived NPF signaling in ovarian somatic cells affects Dpp signaling in GSCs to control their proliferation after mating.

We also analyzed cap cells, which are critical components of the GSC niche. However, *Tkg>NPF*^*RNAi*^ did not change the number of cap cells in virgin or mated female flies (Fig 6C), suggesting that midgut-derived *NPF* does not affect the overall architecture of the niche. This is consistent with observations that found no mating-induced effects on cap cell numbers [14]. Thus, these findings indicate that the NPF-dependent post-mating increase in GSC proliferation is not dependent on the physical size of the GSC niche but rather is regulated through modulation of the Dpp signaling pathway.

### The NPF-dependent increase in GSC number requires ovarian ecdysteroid signaling

Previous studies have revealed that biosynthesis of ecdysone, the major insect steroid hormone, is induced by mating stimuli and that ovarian ecdysteroid transmits its signal directly through the ecdysone receptor (EcR) expressed in the ovarian niche to increase GSC number [14,15,42,44,45]. We therefore examined the relationship between NPF and ecdysteroids in the ovary. Ovarian ecdysteroid levels after mating were not different between control and *Tkg>NPF*^*RNAi*^ animals (S7A Fig), indicating that NPF is not essential for mating-induced ecdysteroid biosynthesis. Consistent with this, exogenous application of 20-hydroxyecdysone—the active ecdysteroid—did not rescue the GSC phenotype of *Tkg>NPF*^*RNAi*^ animals (S7B Fig). However, ex vivo culture experiments revealed that virgin ovaries dissected from animals with knocked down *neverland* (*nvd*), encoding an ecdysteroid biosynthesis enzyme, did not exhibit an increase in GSC number in the presence of synthetic NPF peptide (S7C Fig). In addition, synthetic NPF peptide in ex vivo cultures only induced a minor increase in GSC number in ovaries dissected from *EcR* RNAi animals (S7C Fig). These results suggest that NPF signaling in this context requires ecdysteroid signaling, which possibly interacts with *EcR* and/or downstream signaling components (S7D Fig).

## Discussion

**Fig 7:**
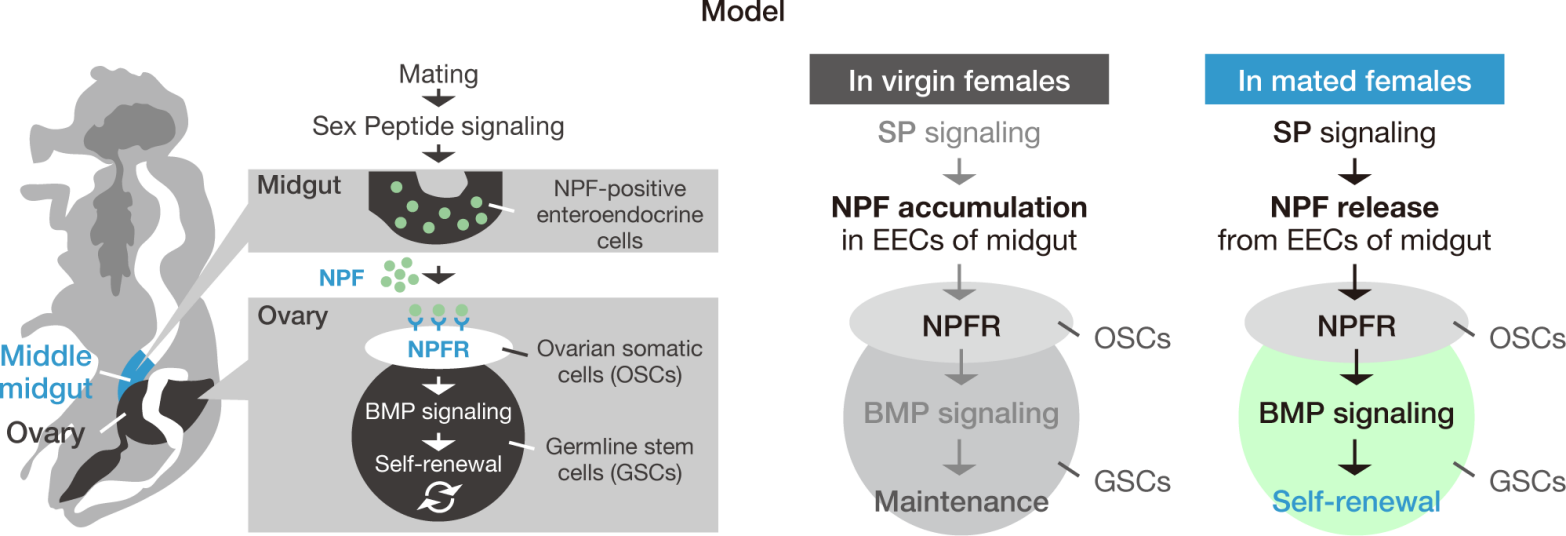
Midgut-derived NPF controls mating-induced GSC proliferation. A model illustrating the mechanism based on this study. SP signaling induces NPF release from midgut EECs. NPF positively controls pMad expression in GSCs to induce GSC proliferation via ovarian NPFR. BMP, bone morphogenetic protein; EEC, enteroendocrine cell; GSC, germline stem cell; NPF, neuropeptide F; NPFR, neuropeptide F receptor; OSC, ovarian somatic cell; pMad, phosphorylated Mad; SP, sex peptide.

Stem cells are maintained by a specialized microenvironment, or niche, that produces local signals. While the importance of these signals is unquestionable, systemic signals from other tissues are also required for stem cell regulation. However, it remains unclear whether and how crucial systemic signals influence stem cell behavior in response to environmental factors. Our present study demonstrated that midgut-derived NPF modulates GSC proliferation in response to mating stimulus. In mated female flies, SP signaling regulates NPF accumulation levels in EECs in the middle midgut. We showed that NPF acts on the ovary to control GSC proliferation via its receptor NPFR. Furthermore, NPF–NPFR signaling positively modulates Dpp signaling in GSCs to support symmetric GSC divisions. Our results reveal a mechanism of interorgan communication between the gut and the ovary that promotes mating-induced activation of gametogenesis. This is the first study to show that a gut-derived factor modulates GSC activity (Fig 7).

In many animals, reproduction involves significant behavioral and physiological shifts in response to mating. In female *Drosophila melanogaster*, post-mating responses result from signals, especially SP, delivered by male seminal fluid during mating. Beyond the previously described SP-dependent post-mating changes [13,33,36,46], our data imply that SP induces the post-mating release of NPF from EECs by silencing *SPR*-positive neurons. However, the molecular and cellular mechanisms by which SP–SPR signaling influences EECs remain unclear. One possibility is that juvenile hormones (JHs) transmit SP-dependent mating signals to the midgut. It has been reported that mating induces JH biosynthesis in an endocrine organ, the corpus allatum, in vivo [29] and that SP stimulates JH biosynthesis in the corpus allatum in vitro [47]. After mating, elevated circulating JH signals are received by the intestinal epithelium, leading to gut remodeling and an increase in gut size, which is required for reproductive success [29]. However, our data indicate that midgut RNAi (in EECs and NPF/Tk/Dh31-positive EECs) against the 2 known genes encoding JH receptors in *Drosophila* (*Methoprene-tolerant* and *germ cell-expressed bHLH-PAS*) does not affect mating-induced GSC proliferation (S4C Fig). Therefore, NPF-dependent control of GSC proliferation appears to be independent of gut JH signaling. It is also unlikely that the midgut directly receives SP because overexpressing *SP* in EECs did not affect NPF accumulation in EECs (S5A and S5B Fig). Consistent with these results, we did not detect anti-SPR immunostaining signals in EECs. It would be interesting to identify the specific humoral factor that conveys the SP-dependent neuronal signal to EECs.

Another possibility is that nutrient intake after mating is the key in releasing NPF from EECs. Because SP signaling is important for the drastic increase in food intake by females after mating [48], it is possible that SP promotes gut NPF secretion indirectly through some nutrient(s) from food. Notably, the presence of amino acids activates calcium signaling in some EECs in the posterior midgut [49]. In addition, sugar is known to affect a subpopulation of EECs, as activin-β in EECs is up-regulated by chronic high-sugar diets and acts on the fat body [50]. Alternatively, metal ions, such as copper ions, would also be interesting candidates because many NPF-positive cells are located in the stomach-like copper cell region that accumulates copper ions [51,52]. Moreover, NPF has already been identified as a regulatory neuropeptide in discriminating nutritional and food-related conditions [53,54]. Future studies examining whether nutrients can affect NPF release from midgut EECs to control mating-induced GSC proliferation will be worthwhile.

Our findings support the notion that ovarian NPFR enhances BMP signaling in GSCs to promote their self-renewal. Transgenic RNAi and overexpression of *NPFR* driven by *tj-GAL4* revealed that NPFR is necessary and sufficient for induction of female GSC proliferation. More remarkably, ex vivo cultures demonstrated that synthetic NPF peptide is sufficient to induce GSC proliferation in dissected ovaries from virgin females. However, we were unable to address which ovarian cell type expresses *NPFR* for controlling GSC proliferation. Even though we showed that *NPFR* transcripts are detected in dissected ovaries (Fig 4C), we failed to observe any clear signals of digoxigenin-labeled *NPFR* RNA probes by in situ hybridization, whereas the same method was successfully applied for detecting *NPFR* mRNA in the head and midgut [38]. We speculate that this may be due to lower amounts of transcript in the ovary than in the head and midgut (Fig 4C).

Nevertheless, our analysis using several cell type–specific *GAL4* drivers suggests that GSC proliferation requires NPFR acting in some combination of escort or follicle cells but not with germ and cap cells. Although cap cells act as the main niche component by producing the short-range Dpp ligand that ensures GSC self-renewal, escort cells also function in the GSC niche by producing Dpp to repress cystoblast differentiation [55,56]. In escort cells, the Hedgehog (Hh) and Janus Kinase (JAK)-Signal Transducer and Activator of Transcription (STAT) signaling pathways are important for GSC maintenance through activating expression of genes encoding BMP ligands [57–59]. Of these 2 pathways, Hh signaling appears particularly relevant in this context. It is well known that activation of Hh signaling results in stabilization and nuclear localization of the transcription factor Cubitus interruptus (Ci) [60]. Conversely, Hh signaling is negatively regulated by proteolysis of Ci following its phosphorylation by several protein kinases, including cAMP-dependent protein kinase (PKA) [61]. Notably, NPF–NPFR signaling negatively regulates adenylyl cyclase activity, leading to down-regulation of cAMP production [38]. Therefore, it is feasible to hypothesize that NPF–NPFR signaling may result in lower cAMP levels and reduced PKA activity, allowing Ci to persist within the nucleus, leading to enhanced Hh signaling in escort cells. Examining genetic interactions between NPF–NPFR signaling and Hh signaling in ovarian somatic cells is currently underway. It is also of interest to detect in vivo cAMP fluctuation by imaging with fluorescence resonance energy transfer (FRET) probes, such as Epac-based FRET sensors [62], to examine which cells actually respond to NPF and observe whether Hh signaling is involved in NPF–NPFR–dependent GSC proliferation.

Although NPF–NPFR signaling positively affects pMad levels in GSCs, it should be noted that there are both positive and negative roles for BMP receptors in NPF–NPFR–dependent regulation of mating-induced GSC proliferation. Our genetic data suggest that the BMP type-I receptor Sax positively regulates mating-induced GSC proliferation, while the other type-I receptor Tkv and the type-II receptor Put negatively regulate it. This complex situation is reminiscent of a recent finding that Tkv plays both positive and negative roles in female GSC proliferation in *Drosophila* [63]. In the latter case, Tkv proteins in escort cells sequester excess cap cell–produced Dpp, thereby reducing Dpp activity. Thus, it is possible that each subtype of BMP receptor may have a unique function in mediating NPF–NPFR signaling in different cell types of the germarium.

In addition to ecdysteroids [44,64] and insulin [10,65], we have identified NPF as a new essential humoral regulator for GSC proliferation and self-renewal. It will be important to investigate whether and how these endocrine signals reciprocally work in GSCs and the GSC niche. While previous studies demonstrated parallel regulation by ecdysone and insulin for GSC proliferation [14,44], our study suggests that NPF–NPFR signaling requires ecdysteroid signaling to control GSC proliferation (S7D Fig). Ecdysteroid signaling is known to be crucial for GSC maintenance that is dependent on intrinsic epigenetic machinery [64,66]. Thus, NPF–NPFR signaling may affect chromatin remodeling in GSCs to control mating-induced GSC proliferation.

The question as to the effective dose of synthetic NPF peptide in adult female flies must be addressed. As shown in Fig 3E, the threshold concentration to induce GSC increase by injection is 3 pM of synthetic NPF peptide, and we estimate a single-fly injection amount of 100 nL. Assuming that the total amount of hemolymph per single female fly is approximately 1 μL, the injected NPF peptide should increase hemolymph NPF titers by approximately 0.3 pM. On the other hand, binding assays with isotope-labeled NPF in mammalian cells shows a half maximal inhibitory concentration (IC_50_) of 65 nM NPF on NPFR [38]. Based on the pharmacological profiles of NPFR, in conjunction with the assumption that the peptide is rapidly degraded in the hemolymph [67], the minimal effective amount of injected synthetic NPF peptide (0.3 pM) seems to be very low. Coupled with a lack of published evidence on the actual chemical characteristics of NPF peptide in hemolymph, the uncertainty as to the exact fate of injected NPF and why our results were reproducible even at concentrations well below the theoretical threshold require further examination. Future studies must address how exogenous NPF peptide behaves in the animal after injection (e.g., which tissues NPF accumulates in or how long it is stable in the hemolymph), whether and how NPF in vivo is biochemically modified or complexed before interaction with the ovary, and the measurement of the actual amount of circulating NPF in virgin and mated females.

In many animal species, NPY has a role in regulating reproduction. In planarians, neuronal neuropeptide Y-8 (NPY-8) and neuropeptide Y receptor Y1 (NPYR-1) signaling regulate germline development, including GSC differentiation [68]. In mammals, administration of NPY results in various effects on luteinizing hormone (LH) and gonadotropin-releasing hormone (GnRH) secretion, either stimulatory or inhibitory. Injection of NPY into ovariectomized and sex steroid-treated rats stimulates secretion of LH and GnRH [69,70]. Conversely, NPY suppresses the gonadotropic axis and delays sexual maturation in intact rats [71–73]. NPY also has a role in coordinating mammalian reproductive function and energy balance [74]. Although several studies have described the role of neuronal NPY signaling on reproduction, the role of intestinal NPY is poorly understood. Therefore, it would be of interest to explore the role of intestinal NPY on reproduction in the context of mammalian nutritional status.

## Materials and methods

### *Drosophila* strains

Flies were raised on cornmeal-yeast-agar medium at 25°C; temperature-sensitive mutants were cultured at 29°C for 1 day prior to performing the assays. *yw* was used as the control strain. The mutant alleles *NPF*^*sk1*^, *NPF*^*sk2*^, *sNPF*^*sk4*^, *sNPF*^*sk8*^, and *NPFR*^*sk8*^ were created in a *yw* background using CRISPR/Cas9 as previously described [16]. The following guide RNA (gRNA) sequences were used: *NPF*, 5ʹ-GCCCTTGCCCTCCTAGCCGC-3ʹ; *sNPF*, 5ʹ-GTTGGGAAAGACGACGGGTC-3ʹ; *NPFR*, 5ʹ-GCGTGCGCTATCTGGACGAC-3ʹ. Breakpoint details of *NPF*^*sk1*^, *NPF*^*sk2*^, and *NPFR*^*sk8*^ are described in Fig 1. The following transgenic and mutant stocks were used: *nSyb-GAL4* (Bloomington 51941), *elav-GAL4* (Bloomington 8765), *386Y-GAL4* (Bloomington 25410), *Tk-gut-GAL4* [20] (gift from Masayuki Miura, the University of Tokyo, Japan), *Myo1A-GAL4* [75] (gift from Kazutaka Akagi, National Center for Geriatrics and Gerontology, Japan), *esg-GAL4* (Bloomington 26816), *SPR-GAL4*::*VP16* (see the section “Establishing the *SPR-GAL4::VP16* strain” below in Materials and methods for details), *tj-GAL4* (Kyoto 104055), *e22c-GAL4* (Kyoto 106609), *c587-GAL4* [76] (gift from Hiroko Sano, Kurume University, Japan), *bab1-GAL4* [77] (gift from Satoru Kobayashi, University of Tsukuba, Japan), *c306-GAL4* (Bloomington 3743), *nos-GAL4* (Kyoto 107748), *UAS-NPF* [17] (gift from Ping Shen, University of Georgia, USA), *UAS-NPFR* (gift from Ping Shen), *UAS-rpr; UAS-hid* (gift from Katja Brückner, University of California San Francisco), *UAS-shi*^*ts1*^ (Bloomington 44222), *dj-GFP/CyO* [78] (Bloomington 5417), *Df(3R)ED10642* (Kyoto 150266), *Df(3R)BSC464* (Bloomington 24968), *dpp*^*hr56*^ [7] (Bloomington 36528), *sax*^*5*^ (Bloomington 8785), *tkv*^*1*^ (Bloomington 427), *put*^*135*^ [7] (Bloomington 3100), and *SP*^*0*^ [79] and *SP*^*Δ130*^ [79] (gifts from Nobuaki Tanaka, Hokkaido University, Japan). Janelia GAL4 stocks [80] were obtained from the Bloomington Drosophila Stock Center: *R41D08-GAL4* (45279), *R42C03-GAL4* (50148), *R46G06-GAL4* (41271), and *R50F10-GAL4* (45998). RNAi constructs targeting *NPFR* (KK107663), *gce* (KK101814), and *Met* (KK100638) were obtained from the Vienna Drosophila Resource Center (VDRC) [81] and lines targeting *NPF* (27237), *NPFR* (*NPFR RNAi-2*; 25939), *Tk* (25800), and *Dh31* (41957) were obtained from the Bloomington TRiP collection [82].

### Behavioral assays

Flies were reared at 25°C and aged for 5 to 7 days. Virgin female flies were mated overnight to *yw* male flies at 25°C (10 males and 5–10 females per vial). For egg-laying assays, individual female flies were transferred to a fresh chamber with cornmeal-yeast-agar medium, after which the eggs were counted manually.

### Immunohistochemistry

Ovaries and midguts were dissected in Grace’s supplemented insect medium (Gibco) and fixed in 4% paraformaldehyde in Grace’s medium for 30 to 60 minutes at room temperature (RT). Fixed samples were washed 3 times in PBS supplemented with 0.1% Triton X-100. After washing, the samples were blocked in blocking solution (PBS with 0.1% Triton X-100 and 0.2% bovine serum albumin [BSA]) for 1 hour at RT and then incubated with a primary antibody in blocking solution at 4°C overnight. Primary antibodies used in this study were mouse anti-Hts 1B1 [83] (1:50; Developmental Studies Hybridoma Bank [DSHB]), rat anti-DE-cadherin DCAD2 [84] (1:50; DSHB), rabbit anti-pH3 (1:1000; Merck Millipore), rabbit monoclonal anti-pMad (1:1000; Abcam), mouse anti-Lamin-C LC28.26 [85] (1:10; DSHB), rat anti-BrdU (1:50; Abcam), rabbit cleaved Dcp-1 (1:100; Cell Signaling Technology), rabbit anti-NPF (1:2000; provided by Ping Shen), rat anti-Vasa (1:1000; DSHB), and Alexa Fluor 546 phalloidin (1:200; Invitrogen). When anti-pMad antibody was used, the immunofluorescent signals were enhanced by Can Get Signal Solution B (ToYoBo). After washing, fluorophore (Alexa Fluor 488 or 546)-conjugated secondary antibodies (Invitrogen) were used at a 1:200 dilution, and the samples were incubated for 2 hours at RT in blocking solution. After another washing step, all samples were mounted in FluorSave reagent (Merck Millipore). For BrdU incorporation, dissected ovaries were incubated in Grace’s medium containing 10 μM BrdU (Sigma-Aldrich) for 1 hour at RT, washed, and then fixed with 4% paraformaldehyde in Grace’s medium for 1 hour. Ovaries were denatured in 2 N HCl for 30 minutes, neutralized in 100 mM borax for 2 minutes, and then immunostained using mouse anti-BrdU (1:50; Abcam). GSC number was determined based on morphology and positioning of their anteriorly anchored spherical spectrosome [44]. Samples were visualized using a Zeiss LSM 700 confocal microscope or Zeiss Axioplan 2. Images were processed using ImageJ software (NIH).

### Quantitative reverse transcription PCR

To quantify mating-induced changes in gene expression, the middle midguts from 5 to 10 adult female flies were dissected. Total RNA was extracted using RNAiso Plus reagent (TaKaRa). cDNA was prepared with ReverTra Ace qPCR RT Master Mix with gDNA Remover (ToYoBo). Quantitative reverse transcription PCR (qRT-PCR) was performed using the Universal SYBR Select Master Mix (Applied Biosystems) with a Thermal Cycler Dice TP800 system (TaKaRa). Serial dilutions of a plasmid containing the open reading frame of each gene were used as standard. The amount of target RNA was normalized to *ribosomal protein 49* (*rp49*) and then relative fold changes were calculated. The following primer pairs were used to measure transcript level: *rp49* forward, 5ʹ-CGGATCGATATGCTAAGCTGT-3ʹ and reverse, 5ʹ-GCGCTTGTTCGATCCGTA-3ʹ; NPF forward, 5ʹ-CTCCGCGAAAGAACGATGTCAACAC-3ʹ and reverse, 5ʹ-CCTCAGGATATCCATCAGCGATCCG-3ʹ; NPFR forward, 5´-GATCCTGTCCAAGTACTGGCCCTAC-3´ and reverse, 5´-ACGATCACCTGATATCTGTCGAAGGC-3´. All qRT-PCR runs were performed in triplicate.

### Establishing the *SPR-GAL4*::*VP16* strain

To prepare the *SPR-GAL4*::*VP16* line, we used a recombineering approach based on previously described methods [86]. To prepare a landing-site cassette, 5′ and 3′ homology arms were amplified from the *GAL4/*terminator gene of *pBPGUw* [87] and were used to flank the positive/negative selectable marker *RpsL-kana* [88], conferring kanamycin resistance and streptomycin sensitivity. *SPR*-specific arms were added to this landing-site cassette by PCR using the following primers: SPR-F, 5ʹ-gaattaaggcagcgccaggggaatccgctcgagaaacccacgtccacgagATGAAGCTACTGTCTTCTATCGAACAAGC-3ʹ and SPR-R, 5ʹ-ttggtgtgcacactaaattatcgatataaacaacaagccatttaacttacGATCTAAACGAGTTTTTAAGCAAACTCACTCCC-3ʹ. These primers contained 50 bases matching the *SPR* locus (in lower case) and sequences corresponding to the *GAL4* and terminator arms that were previously added (in upper case); note the underlined ATG of both *SPR* and *GAL4*. This cassette was recombined into the bacterial artificial chromosome CH321-69P02 [89] (obtained from the Children’s Hospital Oakland Research Institute), containing the *SPR* locus within 88 kb of genomic DNA sequence; recombinants were then selected on kanamycin medium. In a second round of recombination, the landing pad was replaced with full-length *GAL4*::*VP16+terminators* amplified from *pBPGAL4*.*2*::*VP16Uw* [90]; recombinants were identified by streptomycin resistance. Potential regulatory elements in flanking regions, upstream and downstream untranslated regions, and introns remained intact, although regions downstream of the target region are presumably no longer transcribed. The recombined regions were sequenced, and the finished bacterial artificial chromosome was integrated into the *attP40* site by Rainbow Transgenic Flies (Camarillo, CA).

### Fly injection

Fly injection was performed using a previously described technique [91]. NPF peptide amidated at its C-terminus (SNSRPPRKNDVNTMADAYKFLQDLDTYYGDRARVRF-NH_2_) was synthesized by Eurofins Genomics. The synthetic NPF peptide was diluted in PBS to 3 pM. The NPF peptide solution was injected into the thoraces of virgin female flies chilled on ice. Although we could not control the exact amount of peptide solution for fly injection because of limitations imposed by our injection apparatus, we can roughly estimate the amount, which should be 100 nL. Injected flies were transferred into vials with standard fly food. Flies were cultured at RT in vials with or without males for 16 hours. Afterwards, female flies were dissected and immunostained to count GSC number. Synthetic Bm-sNPF peptide (SPSRRLRF-NH_2_) [92] was a gift from Yoshiaki Tanaka (National Agricultural and Food Research Organization, Japan).

### Ex vivo ovary culture

Adult females were cultured on standard medium and dissected in Schneider’s insect medium (Gibco). Approximately 6 ovaries were transferred to a microcentrifuge tube containing 20 μL Schneider’s medium supplemented with 15% fetal calf serum and 0.6% penicillin-streptomycin with the addition of NPF peptide, sNPF peptide, or PBS. Cultures were incubated at RT for 16 hours, and then samples were immunostained to count GSC number.

### Statistical analysis

All experiments were performed independently at least twice. Fluorescence intensity in confocal sections was measured via ImageJ. For NPF quantification, an average of 10 cells were examined for each midgut. For pMad quantification, signal intensity was calculated by measuring the fluorescence intensity in GSCs and CBs, which were costained with anti-Vasa antibody to visualize their cell boundaries. Size of the posterior midgut in confocal sections was measured via ImageJ. Sample sizes were chosen based on the number of independent experiments required for statistical significance and technical feasibility. The experiments were not randomized, and the investigators were not blinded. All statistical analyses were carried out in the “R” software environment [93]. The *P* value is provided in comparison with control and indicated as *P ≤ 0.05, **P ≤ 0.01, ***P ≤ 0.001, and “NS” for nonsignificant (*P* > 0.05).

## Supporting information

S1 Fig NPF localization in the brain and midgut. (A, B) Representative images of adult female brains and midguts immunostained with anti-NPF antibody (green) and monoclonal nc82 (neuropil marker; magenta) or phalloidin (magenta). Anti-NPF signals in neuroendocrine cells (arrowhead) were dramatically reduced by loss of NPF (panel A) or neuronal knockdown of *NPF* (panel B). Anti-NPF signals in midgut EEC were also reduced by loss of NPF (panel A) but not after neuronal knockdown of *NPF* (panel B). (C) Frequency of germaria containing 1, 2, and 3 GSCs (left axis) and the average number of GSCs per germarium (right axis) in virgin (v) and mated (m) female flies. *NPF* RNAi driven by *nSyb-GAL4* (pan-neuronal cells) or *386Y-GAL4* (neuroendocrine cells) had no effect on the mating-induced increase in GSC number. The number of germaria analyzed is shown inside the bars in panel C. For statistical analysis, a Wilcoxon rank sum test was used for panel C. ***P ≤ 0.001. Scale bar = 50 μm in panel A and B. Underlying data can be found in S1 Data. GSC, germline stem cell; NPF, neuropeptide F.
(TIF)

S2 Fig Expression pattern of *Tk-gut-GAL4* driver. (A–C) Representative images of adult female brains and midguts immunostained with anti-NPF antibody (green) or anti-GFP antibody (green) and monoclonal nc82 (neuropil marker; magenta) or phalloidin (magenta). (A, B) *Tkg-GAL4* driver expressed in the brain and VNC but not in the ovary. (C) *NPF* RNAi driven by the *Tkg-GAL4* driver did not reduce anti-NPF levels in the brain and VNC. Scale bar = 50 μm (brain and VNC) and 100 μm (ovary). NPF, neuropeptide F; Tkg-GAL4, Tk-gut-GAL4; VNC, ventral nerve cord.
(TIF)

S3 Fig Expression pattern of several *EEC-GAL4* drivers. Representative images of adult female brains and midguts immunostained with anti-GFP antibody (green), anti-NPF antibody (magenta), or phalloidin (magenta). These *GAL4* drivers were expressed in NPF-positive EECs. Anti-GFP signals were also detected in the brain, VNC, and oviduct, but not the ovary. Scale bar = 50 μm (brain and VNC) and 100 μm (ovary and midgut). AG, accessory gland; EEC, enteroendocrine cell; NPF, neuropeptide F; OV, oviduct; SP, spermatheca; VNC, ventral nerve cord.
(TIF)

S4 Fig Gut NPF is not involved in gut remodeling. (A, B) The number of mitotic cells (panel A) or size (panel B) of the posterior midgut in virgin or mated female flies was not affected by *NPF* RNAi driven by *Tkg-GAL4* (NPF-positive EECs). (C) Frequency of germaria containing 1, 2, and 3 GSCs (left axis) and the average number of GSCs per germarium (right axis) in virgin (v) and mated (m) female flies. *Met* RNAi or *gce* RNAi driven by *Myo1A-GAL4* (ECs) or *Tkg-GAL4* (NPF/Tk/Dh31-positive EECs) had no effect on the mating-induced increase in GSC number. Dots represent the number of mitotic cells in a single middle midgut (panel A) or the diameter of a single posterior midgut (panel B); lines represent the median, and whiskers represent the interquartile range. For statistical analysis, a Wilcoxon rank sum test was used in panel A and C. Student *t* test was used in panel B. ***P ≤ 0.001 and **P ≤ 0.01. Underlying data can be found in S1 Data. Dh31, diuretic hormone 31; EC, enterocyte; EEC, enteroendocrine cell; gce, germ cell-expressed bHLH-PAS; GSC, germline stem cell; Met, Methoprene tolerant; NPF, neuropeptide F; pH3, phospho-histone H3; Tk, Tachykinin; Tkg-GAL4, Tk-gut-GAL4.
(TIF)

S5 Fig SP signaling controls NPF accumulation in midgut EECs. (A, C) Representative images of anti-NPF antibody immunostaining in the middle midgut are shown on the left. Quantification of anti-NPF signal intensity in the middle midgut is shown on the right graph. Anti-NPF signal intensity did not change after overexpressing *membrane-thethered SP* (*mSP*) in EECs (*Tkg-GAL4>mSP*). (B) *NPF* mRNA level did not change in *Tkg-GAL4>mSP* animals. (C) NPF accumulation was reduced by silencing *SPR*-positive neurons (*SPR-GAL4>shi*^*ts1*^), mimicking SP binding to SPR at the restrictive temperature, without mating. (D) Transcript abundance of *NPF* in the middle midgut did not change by this manipulation. Dots represent the relative signal intensity of anti-NPF in a single middle midgut (panel A and C) or relative expression levels of *NPF* in the middle midgut (panel B and D); lines represent the median, and whiskers represent the interquartile range. For statistical analysis, a Wilcoxon rank sum test with Holm’s correction was used for panel A and C. Student *t* test with Holm’s correction was used for panel B and D. ***P ≤ 0.001 and **P ≤ 0.01; NS, nonsignificant (*P* > 0.05). Scale bar = 50 μm in panel A and C. Underlying data can be found in S1 Data. EEC, enteroendocrine cell; mSP, membrane-tethered SP; NPF, neuropeptide F; shi, shibire; SP, sex peptide; SPR, sex peptide receptor; Tkg-GAL4, Tk-gut-GAL4; ts, temperature-sensitive.
(TIF)

S6 Fig Neuronal or intestinal NPFR does not regulate the mating-induced increase in GSC number. (A) Representative images of adult female brains, VNCs, and midguts immunostained with anti-GFP antibody (green) and monoclonal nc82 (neuropil marker; magenta) or phalloidin (magenta) in *c587-GAL4>mCD*::*GFP* or *tj-GAL4>mCD8*::*GFP* females. Both *GAL4* drivers are expressed in the CNS but not the midgut. (B, C) Frequency of germaria containing 1, 2, and 3 GSCs (left axis) and the average number of GSCs per germarium (right axis) in virgin (v) and mated (m) female flies. (B) *NPFR* RNAi driven by *nSyb-GAL4* or *elav-GAL4* (pan-neuronal) did not affect the mating-induced increase in GSC number. (C) *NPFR* RNAi driven by *esg-GAL4* (ISCs and EBs), *Myo1A-GAL4* (ECs), or *Tkg-GAL4* (NPF/Tk/Dh31-positive EECs) had no effect on GSC number after mating. The number of germaria analyzed is shown inside the bars in panel B and C. For statistical analysis, a Wilcoxon rank sum test was used for panel B and C. ***P ≤ 0.001 and **P ≤ 0.01. Scale bar = 50 μm (brain and VNC) or 100 μm (midgut) in panel A. Underlying data can be found in S1 Data. Dh31, diuretic hormone 31; EB, enteroblast; EC, enterocyte; EEC, enteroendocrine cell; GSC, germline stem cell; ISC, intestinal stem cell; NPF, neuropeptide F; NPFR, neuropeptide F receptor; Tk, Tachykinin; VNC, ventral nerve cord.
(TIF)

S7 Fig NPF-dependent increase in GSC number requires ovarian ecdysteroid signaling. (A) Ecdysteroid levels in mated ovaries did not change in *Tkg-GAL4>NPF*^RNAi^ animals. (B, C) Frequency of germaria containing 1, 2, and 3 GSCs (left axis) and the average number of GSCs per germarium (right axis) in virgin (v) and mated (m) female flies. (B) GSC phenotype in *NPF* RNAi animals was not rescued by feeding with the active form of ecdysteroid, 20E. Flies were fed on standard cornmeal-yeast-agar yeast medium mixed with a solution of 20E in ethanol, resulting in a final concentration of 0.1 mM 20E. (C) Ovarian knockdown of *nvd* (*c587-GAL4>nvd*^RNAi^) or *EcR* (*c587-GAL4*>*EcR*^*RNAi*^) blocked the NPF-induced increase in GSC number in ex vivo ovary cultures. (D) A model illustrating GSC regulation by NPF and ecdysteroid. NPF-dependent increase in GSC number requires ovarian ecdysteroid signaling. Dots represent ovarian ecdysteroid levels of female flies (panel A); lines represent the median, and whiskers represent the interquartile range. The number of germaria analyzed is shown inside the bars in panel B and C. For statistical analysis, Student *t* test was used for panel A, and a Wilcoxon rank sum test with Holm’s correction was used for panel B and C. ***P ≤ 0.001 and *P ≤ 0.05; NS, nonsignificant (*P* > 0.05). Underlying data can be found in S1 Data. 20E, 20-hydroxyecdysone; BMP, bone morphogenetic protein; EcR, ecdysone receptor; GSC, germline stem cell; NPF, neuropeptide F; NPFR, neuropeptide F receptor; nvd, neverland; Tkg-GAL4, Tk-gut-GAL4.
(TIF)

S1 Data Underlying data for main and supporting figures. In this file, separate worksheets contain the data used in each figure panel as indicated.
(XLSX)

## Abbreviations

BMP: bone morphogenetic protein
BrdU: bromodeoxyuridine
BSA: bovine serum albumin
Cas9: CRISPR-associated protein 9
Ci: Cubitus interruptus
CRISPR: Clustered Regularly Interspaced Short Palindromic Repeats
Dcp-1: Death caspase-1
Dh31: diuretic hormone 31
Dilp2: *Drosophila* insulin-like peptide 2
Dpp: Decapentaplegic
DSHB: Developmental Studies Hybridoma Bank
EcR: ecdysone receptor
EEC: enteroendocrine cell
FRET: fluorescence resonance energy transfer
GnRH: gonadotropin-releasing hormone
gRNA: guide RNA
GSC: germline stem cell
Hh: Hedgehog
hid: head involution defective
IC_50_: the half maximal inhibitory concentration
JAK-STAT: Janus Kinase-Signal Transducer and Activator of Transcription
JH: juvenile hormone
LH: luteinizing hormone
mSP: membrane-tethered SP
NPF: neuropeptide
NPFR: neuropeptide F receptor
NPY: neuropeptide
NPYR-1: neuropeptide Y receptor Y1
nvd: neverland
PBS: phosphate-buffered saline
pH3: phospho-histone H3
PKA: cAMP-dependent protein kinase
pMad: phosphorylated Mad
ppk: pickpocket
Put: Punt
qRT-PCR: quantitative reverse transcription PCR
RNAi: RNA interference
rp49: ribosomal protein 49
rpr: reaper
RT: room temperature
Sax: Saxphone
sNPF: short neuropeptide F precursor
SP: sex peptide
SPR: sex peptide receptor
Tk: Tachykinin
Tkv: Thickveins
