# Supplementary figures and images for: Midgut-derived neuropeptide F controls germline stem cell proliferation in a mating-dependent manner
Source: PLoS Biol. 2018 Sep 24;16(9):e2005004. doi: 10.1371/journal.pbio.2005004 (PMC6152996; doi:10.1371/journal.pbio.2005004)

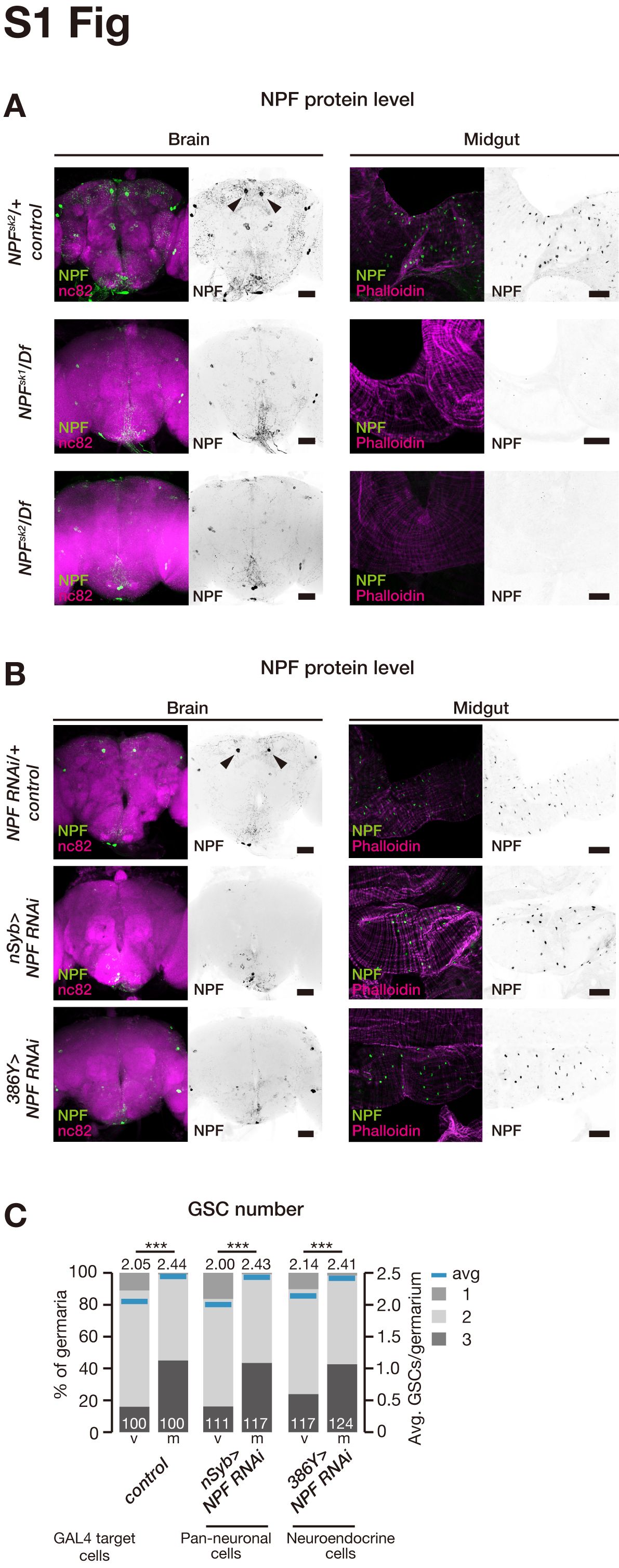

Supplement: S1 Fig — (A, B) Representative images of adult female brains and midguts immunostained with anti-NPF antibody (green) and monoclonal nc82 (neuropil marker; magenta) or phalloidin (magenta). Anti-NPF signals in neuroendocrine cells (arrowhead) were dramatically reduced by loss of NPF (panel A) or neuronal knockdown of NPF (panel B). Anti-NPF signals in midgut EEC were also reduced by loss of NPF (panel A) but not after neuronal knockdown of NPF (panel B). (C) Frequency of germaria containing 1, 2, and 3 GSCs (left axis) and the average number of GSCs per germarium (right axis) in virgin (v) and mated (m) female flies. NPF RNAi driven by nSyb-GAL4 (pan-neuronal cells) or 386Y-GAL4 (neuroendocrine cells) had no effect on the mating-induced increase in GSC number. The number of germaria analyzed is shown inside the bars in panel C. For statistical analysis, a Wilcoxon rank sum test was used for panel C. ***P ≤ 0.001. Scale bar = 50 μm in panel A and B. Underlying data can be found in S1 Data. GSC, germline stem cell; NPF, neuropeptide F. (TIF) [file pbio.2005004.s001.tif]

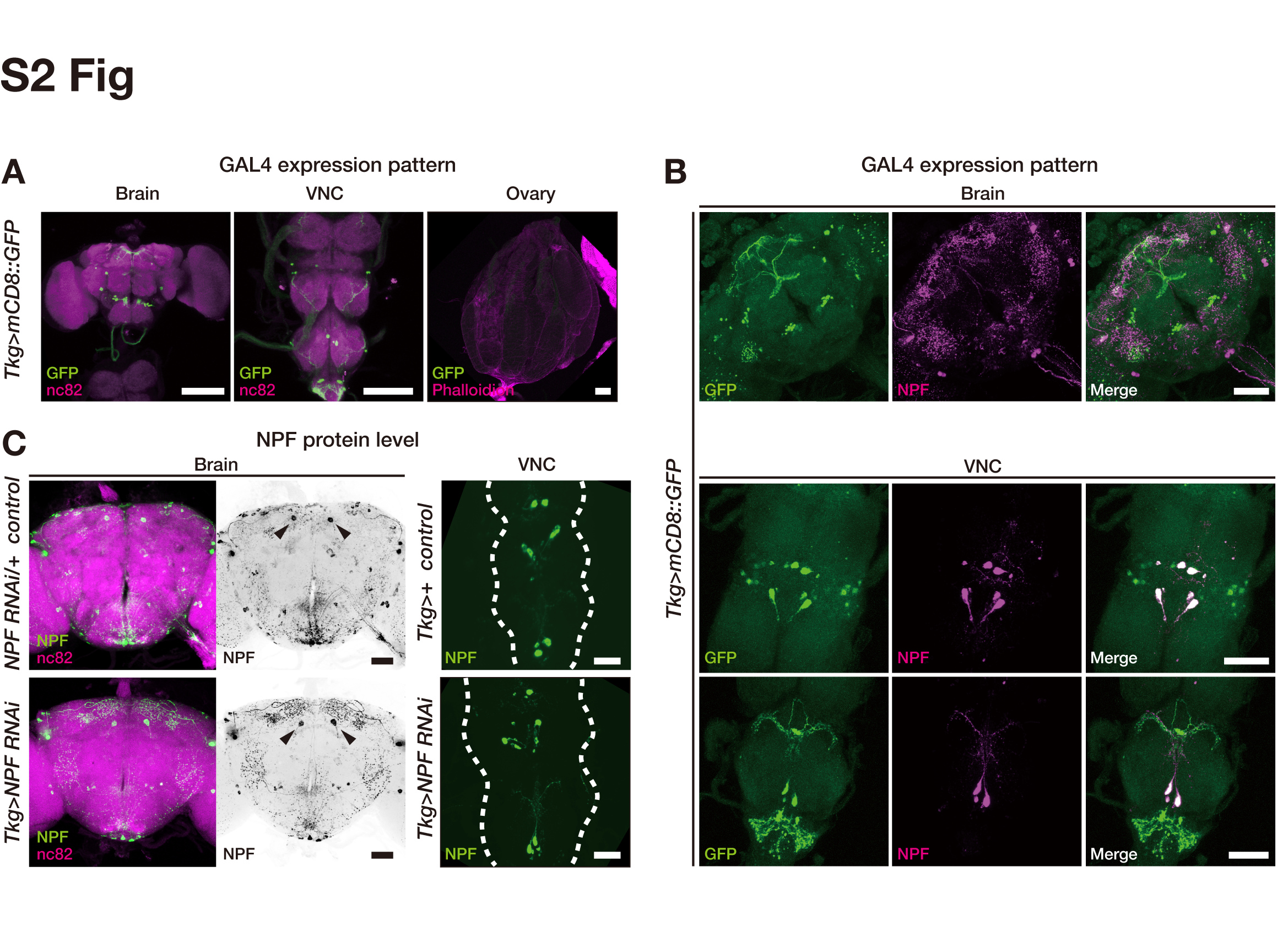

Supplement: S2 Fig — (A–C) Representative images of adult female brains and midguts immunostained with anti-NPF antibody (green) or anti-GFP antibody (green) and monoclonal nc82 (neuropil marker; magenta) or phalloidin (magenta). (A, B) Tkg-GAL4 driver expressed in the brain and VNC but not in the ovary. (C) NPF RNAi driven by the Tkg-GAL4 driver did not reduce anti-NPF levels in the brain and VNC. Scale bar = 50 μm (brain and VNC) and 100 μm (ovary). NPF, neuropeptide F; Tkg-GAL4, Tk-gut-GAL4; VNC, ventral nerve cord. (TIF) [file pbio.2005004.s002.tif]

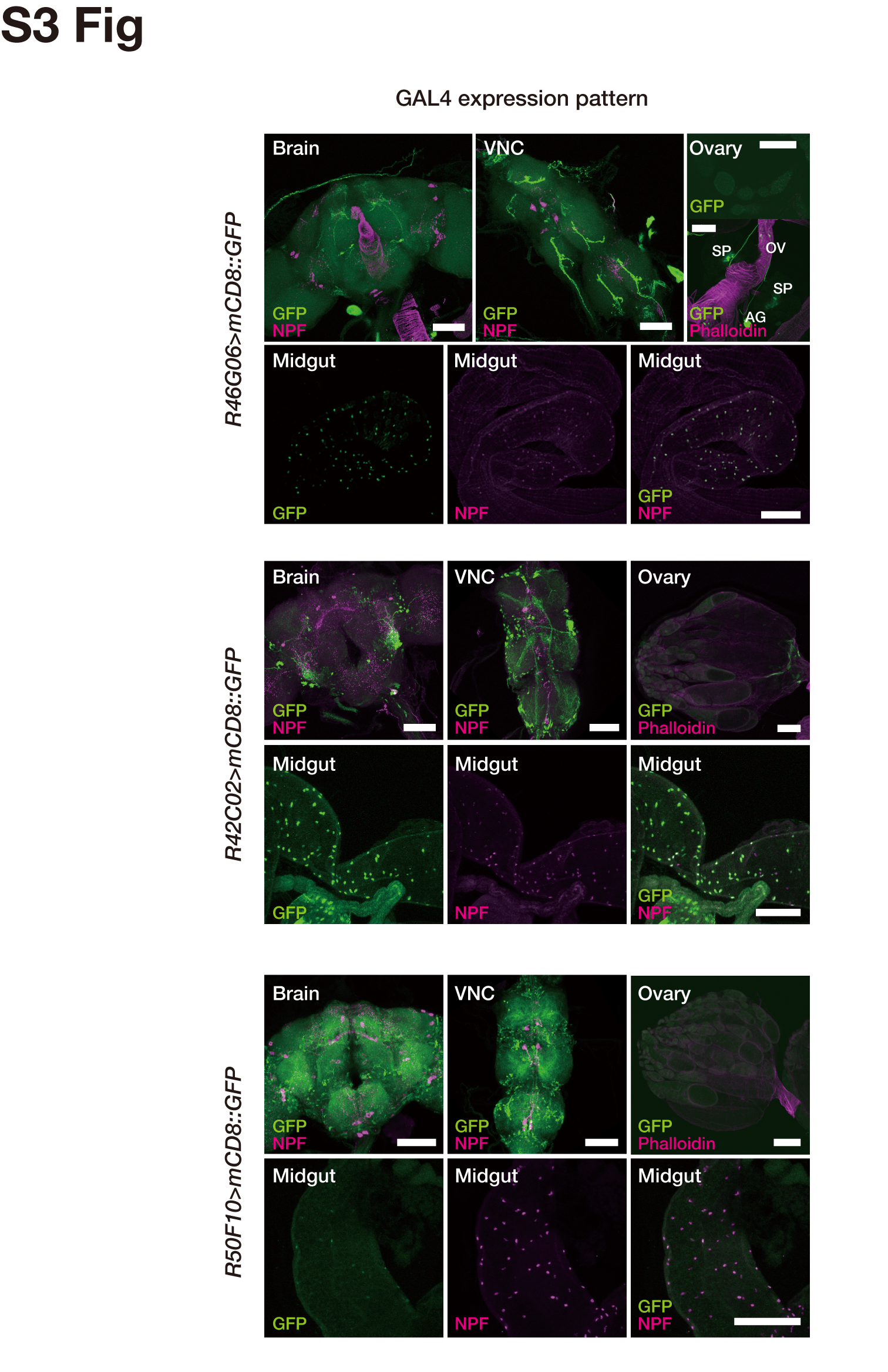

Supplement: S3 Fig — Representative images of adult female brains and midguts immunostained with anti-GFP antibody (green), anti-NPF antibody (magenta), or phalloidin (magenta). These GAL4 drivers were expressed in NPF-positive EECs. Anti-GFP signals were also detected in the brain, VNC, and oviduct, but not the ovary. Scale bar = 50 μm (brain and VNC) and 100 μm (ovary and midgut). AG, accessory gland; EEC, enteroendocrine cell; NPF, neuropeptide F; OV, oviduct; SP, spermatheca; VNC, ventral nerve cord. (TIF) [file pbio.2005004.s003.tif]

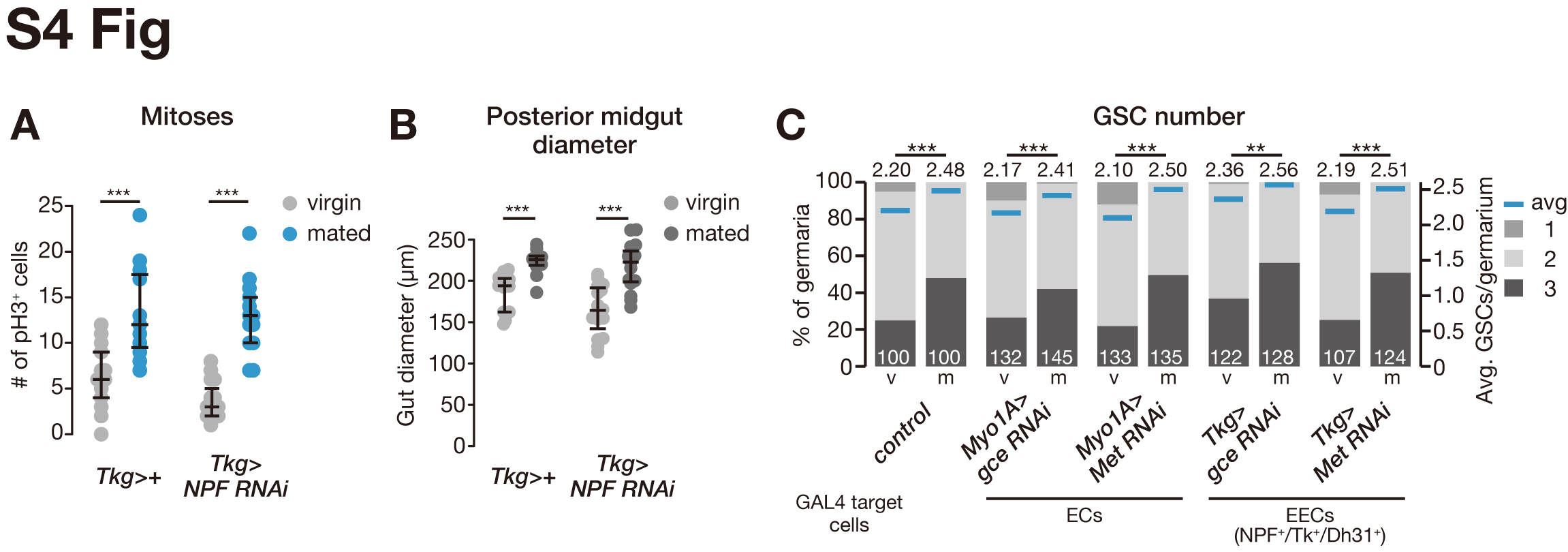

Supplement: S4 Fig — (A, B) The number of mitotic cells (panel A) or size (panel B) of the posterior midgut in virgin or mated female flies was not affected by NPF RNAi driven by Tkg-GAL4 (NPF-positive EECs). (C) Frequency of germaria containing 1, 2, and 3 GSCs (left axis) and the average number of GSCs per germarium (right axis) in virgin (v) and mated (m) female flies. Met RNAi or gce RNAi driven by Myo1A-GAL4 (ECs) or Tkg-GAL4 (NPF/Tk/Dh31-positive EECs) had no effect on the mating-induced increase in GSC number. Dots represent the number of mitotic cells in a single middle midgut (panel A) or the diameter of a single posterior midgut (panel B); lines represent the median, and whiskers represent the interquartile range. For statistical analysis, a Wilcoxon rank sum test was used in panel A and C. Student t test was used in panel B. ***P ≤ 0.001 and **P ≤ 0.01. Underlying data can be found in S1 Data. Dh31, diuretic hormone 31; EC, enterocyte; EEC, enteroendocrine cell; gce, germ cell-expressed bHLH-PAS; GSC, germline stem cell; Met, Methoprene tolerant; NPF, neuropeptide F; pH3, phospho-histone H3; Tk, Tachykinin; Tkg-GAL4, Tk-gut-GAL4. (TIF) [file pbio.2005004.s004.tif]

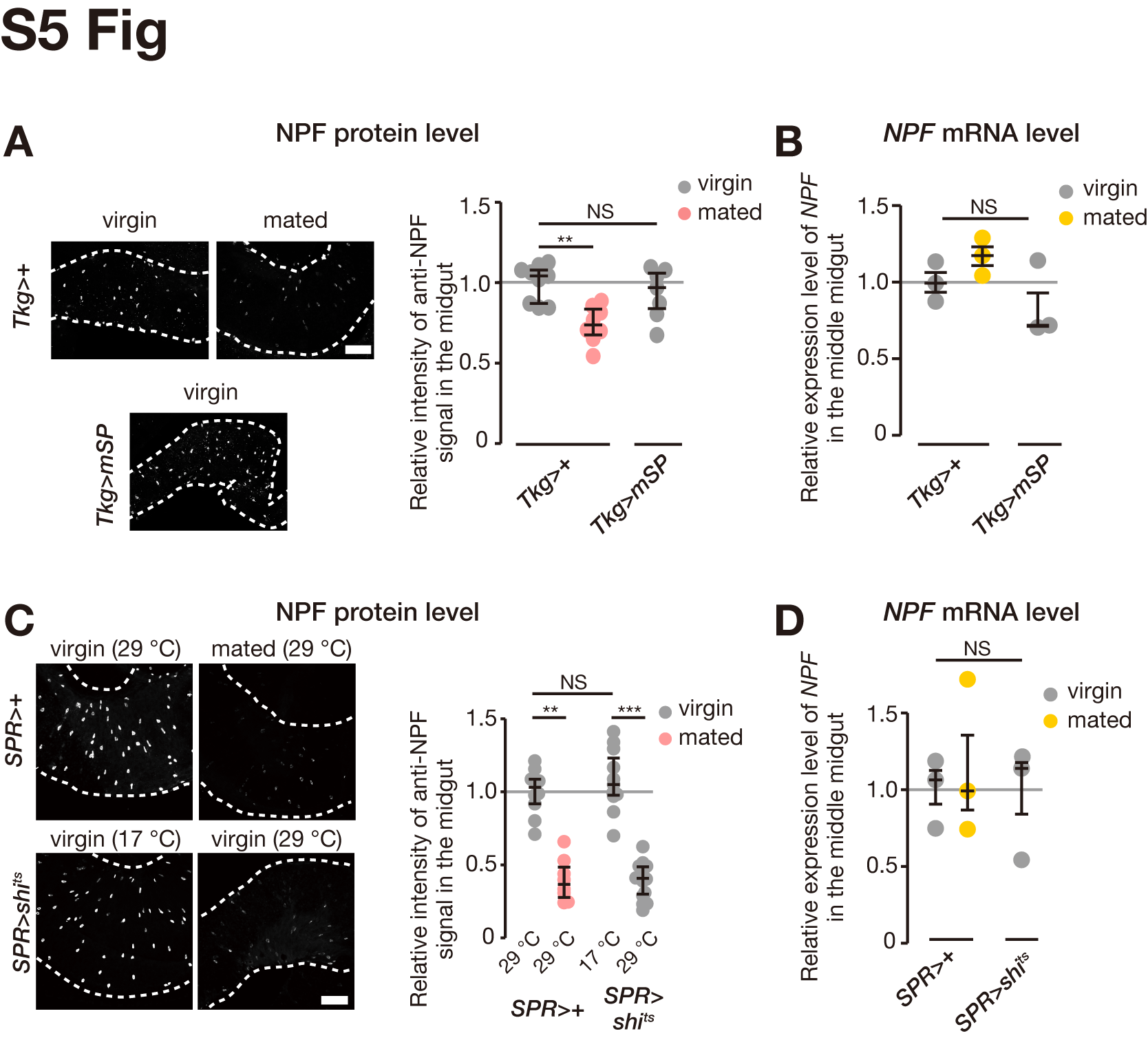

Supplement: S5 Fig — (A, C) Representative images of anti-NPF antibody immunostaining in the middle midgut are shown on the left. Quantification of anti-NPF signal intensity in the middle midgut is shown on the right graph. Anti-NPF signal intensity did not change after overexpressing membrane-thethered SP (mSP) in EECs (Tkg-GAL4>mSP). (B) NPF mRNA level did not change in Tkg-GAL4>mSP animals. (C) NPF accumulation was reduced by silencing SPR-positive neurons (SPR-GAL4>shits1), mimicking SP binding to SPR at the restrictive temperature, without mating. (D) Transcript abundance of NPF in the middle midgut did not change by this manipulation. Dots represent the relative signal intensity of anti-NPF in a single middle midgut (panel A and C) or relative expression levels of NPF in the middle midgut (panel B and D); lines represent the median, and whiskers represent the interquartile range. For statistical analysis, a Wilcoxon rank sum test with Holm’s correction was used for panel A and C. Student t test with Holm’s correction was used for panel B and D. ***P ≤ 0.001 and **P ≤ 0.01; NS, nonsignificant (P > 0.05). Scale bar = 50 μm in panel A and C. Underlying data can be found in S1 Data. EEC, enteroendocrine cell; mSP, membrane-tethered SP; NPF, neuropeptide F; shi, shibire; SP, sex peptide; SPR, sex peptide receptor; Tkg-GAL4, Tk-gut-GAL4; ts, temperature-sensitive. (TIF) [file pbio.2005004.s005.tif]

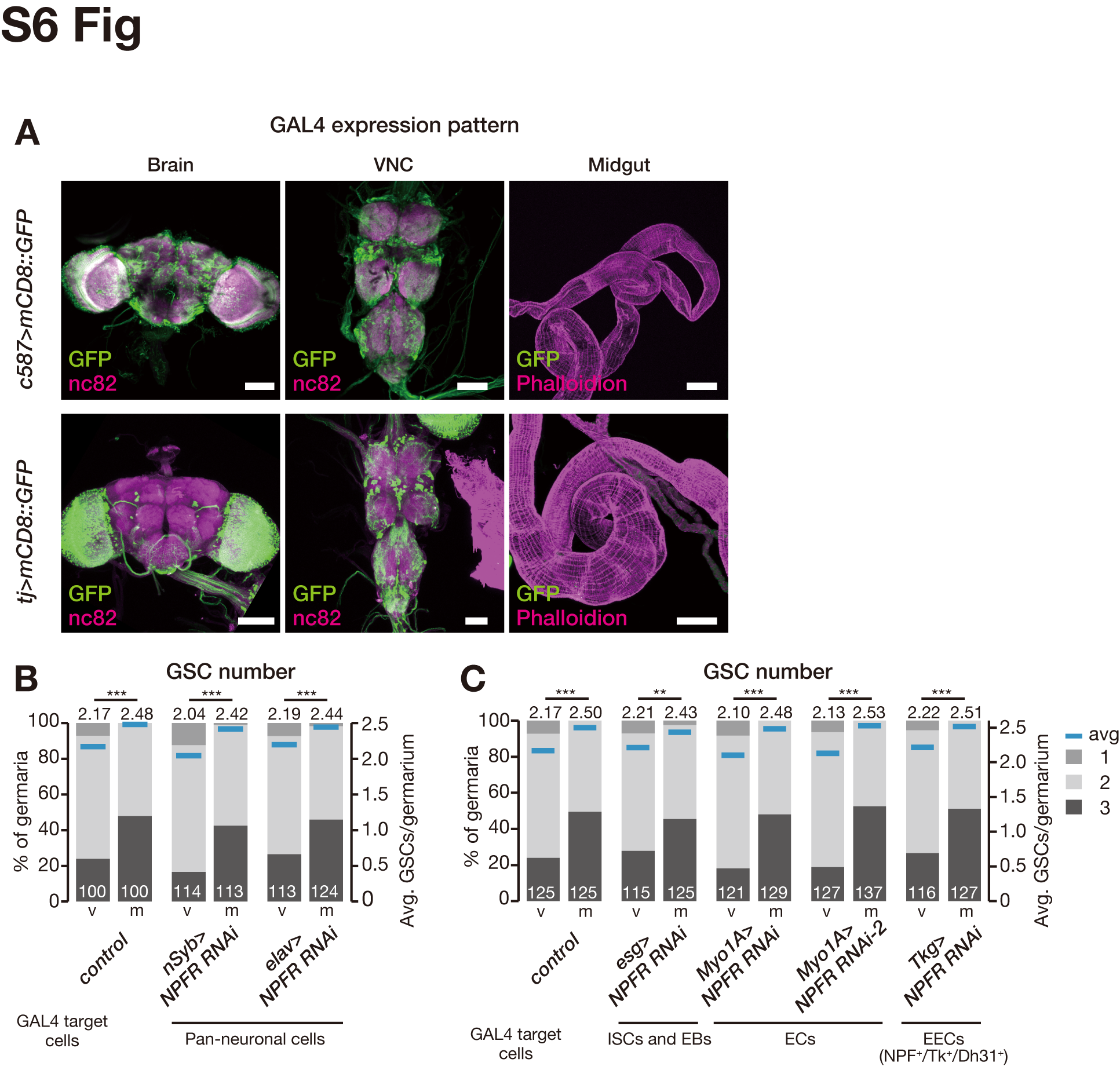

Supplement: S6 Fig — (A) Representative images of adult female brains, VNCs, and midguts immunostained with anti-GFP antibody (green) and monoclonal nc82 (neuropil marker; magenta) or phalloidin (magenta) in c587-GAL4>mCD::GFP or tj-GAL4>mCD8::GFP females. Both GAL4 drivers are expressed in the CNS but not the midgut. (B, C) Frequency of germaria containing 1, 2, and 3 GSCs (left axis) and the average number of GSCs per germarium (right axis) in virgin (v) and mated (m) female flies. (B) NPFR RNAi driven by nSyb-GAL4 or elav-GAL4 (pan-neuronal) did not affect the mating-induced increase in GSC number. (C) NPFR RNAi driven by esg-GAL4 (ISCs and EBs), Myo1A-GAL4 (ECs), or Tkg-GAL4 (NPF/Tk/Dh31-positive EECs) had no effect on GSC number after mating. The number of germaria analyzed is shown inside the bars in panel B and C. For statistical analysis, a Wilcoxon rank sum test was used for panel B and C. ***P ≤ 0.001 and **P ≤ 0.01. Scale bar = 50 μm (brain and VNC) or 100 μm (midgut) in panel A. Underlying data can be found in S1 Data. Dh31, diuretic hormone 31; EB, enteroblast; EC, enterocyte; EEC, enteroendocrine cell; GSC, germline stem cell; ISC, intestinal stem cell; NPF, neuropeptide F; NPFR, neuropeptide F receptor; Tk, Tachykinin; VNC, ventral nerve cord. (TIF) [file pbio.2005004.s006.tif]

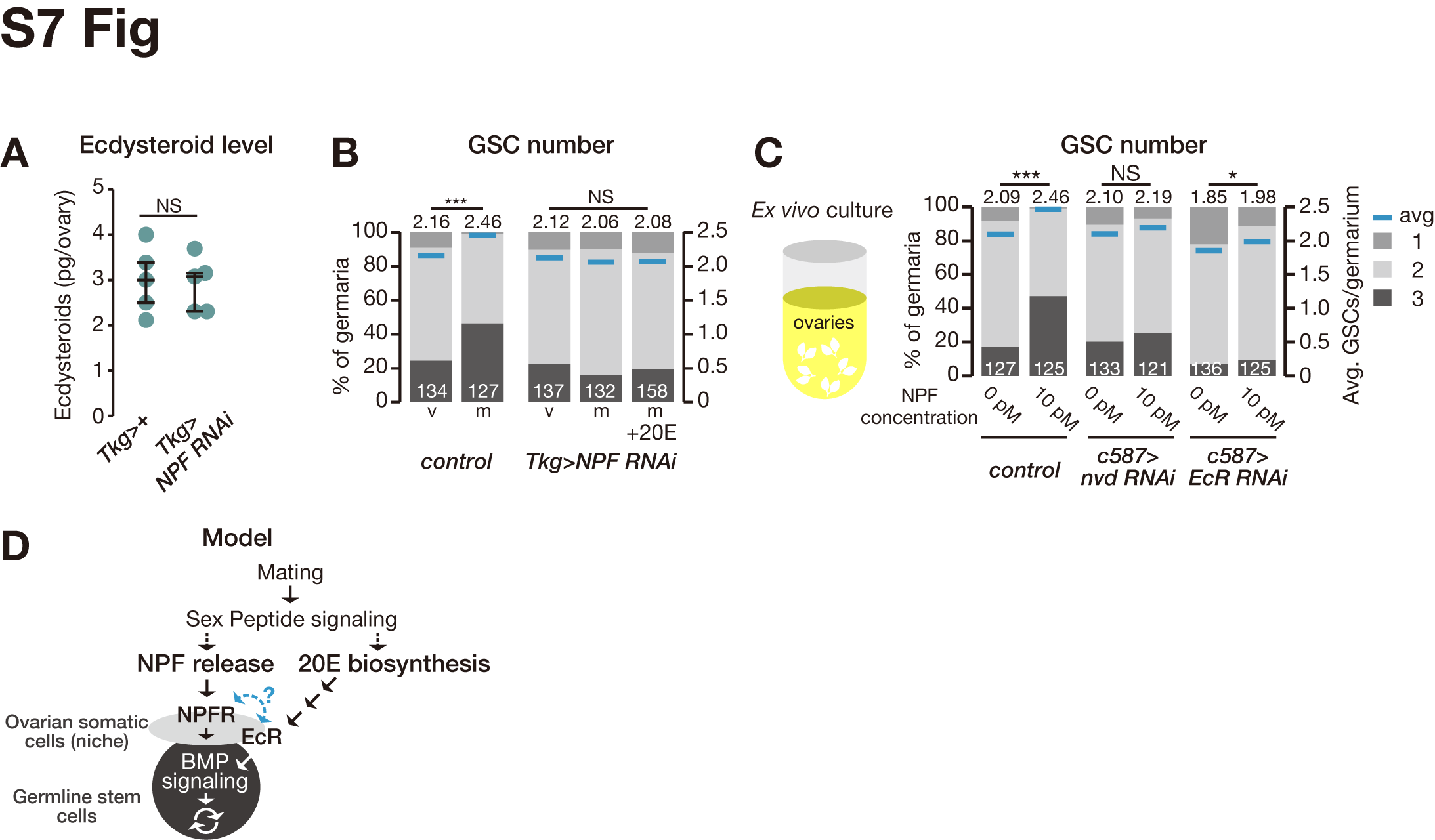

Supplement: S7 Fig — (A) Ecdysteroid levels in mated ovaries did not change in Tkg-GAL4>NPFRNAi animals. (B, C) Frequency of germaria containing 1, 2, and 3 GSCs (left axis) and the average number of GSCs per germarium (right axis) in virgin (v) and mated (m) female flies. (B) GSC phenotype in NPF RNAi animals was not rescued by feeding with the active form of ecdysteroid, 20E. Flies were fed on standard cornmeal-yeast-agar yeast medium mixed with a solution of 20E in ethanol, resulting in a final concentration of 0.1 mM 20E. (C) Ovarian knockdown of nvd (c587-GAL4>nvdRNAi) or EcR (c587-GAL4>EcRRNAi) blocked the NPF-induced increase in GSC number in ex vivo ovary cultures. (D) A model illustrating GSC regulation by NPF and ecdysteroid. NPF-dependent increase in GSC number requires ovarian ecdysteroid signaling. Dots represent ovarian ecdysteroid levels of female flies (panel A); lines represent the median, and whiskers represent the interquartile range. The number of germaria analyzed is shown inside the bars in panel B and C. For statistical analysis, Student t test was used for panel A, and a Wilcoxon rank sum test with Holm’s correction was used for panel B and C. ***P ≤ 0.001 and *P ≤ 0.05; NS, nonsignificant (P > 0.05). Underlying data can be found in S1 Data. 20E, 20-hydroxyecdysone; BMP, bone morphogenetic protein; EcR, ecdysone receptor; GSC, germline stem cell; NPF, neuropeptide F; NPFR, neuropeptide F receptor; nvd, neverland; Tkg-GAL4, Tk-gut-GAL4. (TIF) [file pbio.2005004.s007.tif]
